# Functional Activity of Isoform 2 of Human eRF1

**DOI:** 10.3390/ijms25147997

**Published:** 2024-07-22

**Authors:** Alexey Shuvalov, Alexandr Klishin, Nikita Biziaev, Ekaterina Shuvalova, Elena Alkalaeva

**Affiliations:** 1 Engelhardt Institute of Molecular Biology, The Russian Academy of Sciences, 119991 Moscow, Russia; laursen1243@mail.ru (A.S.);; 2 Center for Precision Genome Editing and Genetic Technologies for Biomedicine, Engelhardt Institute of Molecular Biology, The Russian Academy of Sciences, 119991 Moscow, Russia

**Keywords:** eRF1, eRF3, translation termination, ribosome, readthrough

## Abstract

Eukaryotic release factor eRF1, encoded by the *ETF1* gene, recognizes stop codons and induces peptide release during translation termination. *ETF1* produces several different transcripts as a result of alternative splicing, from which two eRF1 isoforms can be formed. Isoform 1 codes well-studied canonical eRF1, and isoform 2 is 33 amino acid residues shorter than isoform 1 and completely unstudied. Using a reconstituted mammalian in vitro translation system, we showed that the isoform 2 of human eRF1 is also involved in translation. We showed that eRF1iso2 can interact with the ribosomal subunits and pre-termination complex. However, its codon recognition and peptide release activities have decreased. Additionally, eRF1 isoform 2 exhibits unipotency to UGA. We found that eRF1 isoform 2 interacts with eRF3a but stimulated its GTPase activity significantly worse than the main isoform eRF1. Additionally, we studied the eRF1 isoform 2 effect on stop codon readthrough and translation in a cell-free translation system. We observed that eRF1 isoform 2 suppressed stop codon readthrough of the uORFs and decreased the efficiency of translation of long coding sequences. Based on these data, we assumed that human eRF1 isoform 2 can be involved in the regulation of translation termination. Moreover, our data support previously stated hypotheses that the GTS loop is important for the multipotency of eRF1 to all stop codons. Whereas helix α1 of the N-domain eRF1 is proposed to be involved in conformational rearrangements of eRF1 in the A-site of the ribosome that occur after GTP hydrolysis by eRF3, which ensure hydrolysis of peptidyl-tRNA at the P site of the ribosome.

## 1. Introduction

Eukaryotic release factor 1 (eRF1) is one of the two main factors involved in translation termination in eukaryotes [1,2,3]. During translation termination, one of the three stop codons (UAA, UAG, or UGA) is recognized by eRF1 bound with the eukaryotic release factor 3 (eRF3) in the ribosomal A site [3,4,5,6,7,8,9]. Stop codon recognition induces GTP hydrolysis performed by eRF3 [2,3,10,11]. Then conformational rearrangement of eRF1 occurs, and the GGQ motif of eRF1 is accommodated in the peptidyl transferase center of the ribosome [12,13,14,15]. In the peptidyl transferase center, eRF1 induces peptidyl tRNA hydrolysis and the release of nascent polypeptide [1,2,3].

eRF1 consists of three domains [16,17,18,19,20,21]: the N-terminal domain (N) is responsible for stop codon recognition [4,5,6,7,8,9,22], the middle domain (M) contains a conservative GGQ motif, inducing peptidyl-tRNA hydrolysis in the peptidyl transferase center [19,23,24,25] and binds with eRF3 [12], and the C-terminal domain (C) binds with eRF3 [10,12]. Several conserved motifs and amino acid residues are involved in stop codon recognition: the NIKS motif (61–64 residues in human eRF1) recognizing the first U of the stop codon [13,14,15,26], E55, the YxCxF motif (125–129 residues in human eRF1), and the GTS loop (31–33 residues in human eRF1) recognizing the second and third purines [5,6,8,13,14,15]. Additionally, it was shown that the fourth nucleotide of the stop codon is involved in stop codon recognition, stacking against G626 of 18S rRNA [14,15]. During stop codon recognition, eRF1 and the ribosome form a pocket in the A site where mRNA adopts the U-turn structure [14,15] detectable by the toe-printing analysis as 1–2 nt shift of the ribosome to the 3′-end of mRNA [3,8,27,28].

eRF1 interacts with the C-terminal domain of eRF3 during translation termination [2,29]. The GTPase activity of eRF3 is triggered by interaction with eRF1 and the ribosome [2,10,11]. During GTP hydrolysis, both proteins change their conformations, and eRF1 acquires the ability to induce peptidyl-tRNA hydrolysis [13,14]. It was shown that eRF3 interacts with the poly(A)-binding protein (PABP), which facilitates the loading of the release factors into the ribosome [30,31]. The role of several additional proteins in translation termination was revealed [32]. Thus, it was demonstrated that ribosome recycling factor ABCE1 (Rli1 in yeast) binds with the C domain of eRF1 in the ribosome and increases peptide release [33,34,35,36,37]; helicase DDX19 (Dbp5 in yeasts), involved in mRNA transport from the nucleus, participates in translation termination [38,39,40,41]; and eukaryotic translation initiation factor eIF3j also facilitates the loading of release factors into the ribosome via binding with the eRF1–eRF3–GTP complex [42,43,44].

**Figure 1:**
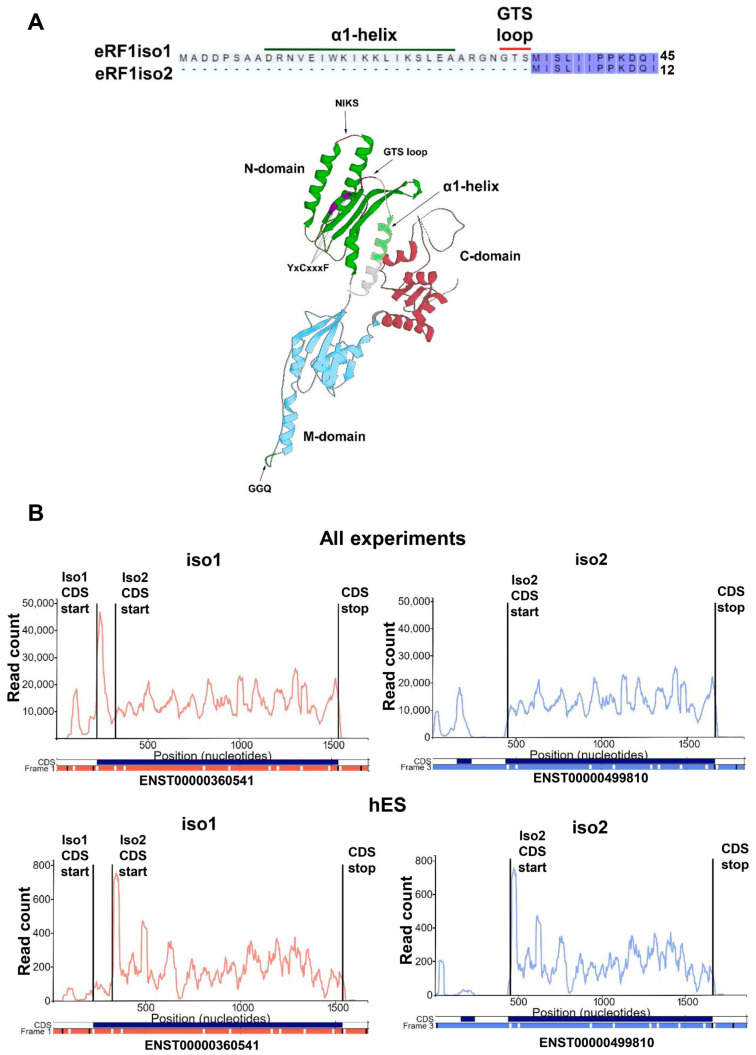
Expression of isoforms of human eRF1. (**A**) Amino acid sequence and structure comparison of isoforms of human eRF1. Isoform 2 of eRF1 is truncated for the first 33 amino acid residues, which include a α1-helix and important for stop codon recognition GTS motif. PDB: 1DT9. (**B**) Visualization of data on ribosomal profiling of human eRF1 transcripts (ENST00000360541–iso1; ENST00000499810–iso2) in the Trips-viz browser (URL (accessed on 26 May 2024): https://trips.ucc.ie/) [45,46]. Combined data show translation of the first 33 codons, unique to isoform 1, while the expression level of isoform 2 cannot be assessed. However, analysis of ribosomal profiling of human embryonic stem (hES) cells shows a reduced density of ribosome distribution in the first 33 codons and a significantly higher level of representation of ribosomal complexes on the common sequence for two isoforms.

The regulation of eRF1 expression has been poorly studied. Human eRF1 is encoded by the *ETF1* gene located on chromosome 5. Six transcriptional variants of the *ETF1* gene are described in the GenBank database. These variants result from alternative splicing, encoding eRF1 isoforms. Human eRF1 main isoform 1 (eRF1iso1, Uniprot ID P62495-1) is encoded by transcript 1 (NCBI RefSeq NM_004730.4; ENST00000360541, URL (accessed on 26 May 2024): www.ensembl.org/index.html), which is widely represented in all tissues. Human eRF1 isoform 2 (eRF1iso2, Uniprot ID P62495-2) is encoded by transcripts 2, 4, 5, and 6 (NM_001256302.2, NM_001291974.2, NM_001291975.2, NM_001364160.2; ENST00000360541). Transcript 3 (NM_001282185.2; ENST00000503014) encodes eRF1 isoform 3 (eRF1iso3, Uniprot ID B7Z7P8), which is not translated in cells (discussed below). It should be noted that the second start codon within the reading frame of the coding sequences of transcripts 1 and 3 corresponds to the first for eRF1iso2. These transcripts may also be capable of producing eRF1iso2 in the case of leaky scanning. The resulting peptide from eRF1iso2 is shorter than the polypeptide encoded by eRF1iso1 for 33 amino acid residues (Figure 1A). Thus, eRF1iso2 does not contain the GTS loop and helix α1 of the N-domain of eRF1 (Figure 1A) [8,13,14,15,21], which may reduce the activity of this protein. However, all accessible data about the activity of eRF1 in translation are available only for eRF1iso1, while eRF1iso2 is completely unstudied.

Since the function of eRF1iso2 of human eRF1 is obscure, we focused our study on the activity of this protein in translation termination. We obtained recombinant eRF1iso2 and examined its activity in stop codon recognition, peptide release, and stimulation of the GTPase activity of eRF3a. Additionally, we studied the regulation of stop codon readthrough and translation efficiency by this protein. Moreover, we identified binding partners of eRF1iso2 that allowed us to suppose a model of regulation of translation termination in uORFs. Additionally, our data allow us to draw conclusions regarding the role of the GTS motif and helix α1 of the N-domain of eRF1 in translation termination.

## 2. Results

### 2.1. Isoform 2 of Human eRF1 Is Found in the Ribosomal Subunits

According to ribosomal profiling data, the isoform 2 of human eRF1, ENST00000499810 (URL (accessed on 26 May 2024): www.ensembl.org/index.html), may be translated (Figure 1B). Since the main parts of the coding sequences of two isoforms of eRF1 do not differ, their ribosomal profiles are similar. All available ribosome profiling experiment data analysis showed translation of the first 33 codons unique to the eRF1iso1, while the expression level of eRF1iso2 cannot be determined on the basis of the summarized data. However, ribosomal profiling data analysis of human embryonic stem (hES) cells [47] showed a reduced density of ribosome distribution in the first 33-codon area and a significantly higher level of representation of ribosomal complexes on the rest of the coding sequence. Consequently, in that line of hES cells, eRF1iso2 is predominantly translated. Interestingly, the human eRF1 isoform 3 (eRF1iso3), annotated on the basis of transcriptomic data in Uniprot (ID B7Z7P8), is not translated accordingly to Ribo-seq data (Figure S1). All available ribosomal profiling data of human cell lines show a complete absence of ribosomes in the area corresponding to the first codons of isoform 3 of eRF1. Ribosomes are present only on the part of the transcript common for all isoforms and corresponding to the translation of eRF1iso1 and/or eRF1iso2. It is worth noting that the distinct 5’UTR transcripts encoding eRF1iso1 and eRF1iso2 are also characterized by the presence of ribosomes, the distribution density of which corresponds to the unique uORFs located there (Figure 1B), indicating independent translation of these transcripts. This is not observed for the transcript encoding the hypothetical eRF1iso3 (ENST00000503014) despite the presence of a uORF (Figure S1). Perhaps this transcript is not translated.

**Figure 2:**
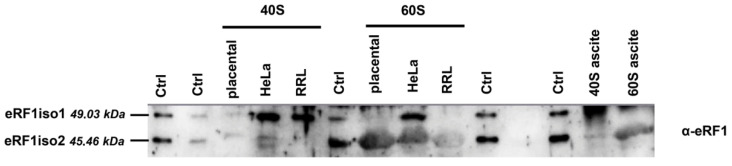
eRF1iso2 bound to different ribosomes. Western blot analysis of ribosomal subunits isolated from placenta, HeLa, RRL, and Krebs-2 lysate. Antibodies raised against eRF1 (sc-365686) were used for detection. Ctrl—mixture of recombinant eRF1iso1 and eRF1iso2. The first and second lanes contain recombinant eRF1iso1 and eRF1iso2, 0.005 and 0.0025 pmol, respectively. All other control lanes (6, 10, 13) contain 0.005 pmol of recombinant eRF1iso1 and eRF1iso2.

To detect eRF1iso2 in the human translation apparatus, we performed Western blot hybridization of 40S and 60S ribosomal subunits purified from different sources with antibodies against the C-terminal domain of eRF1. Additionally, we constructed vectors for expression in bacteria of this form of human eRF1 and purified the recombinant protein. As eRF1iso2 is shorter than eRF1iso1 by 33 amino acid residues, we managed to separate them on SDS-PAAG (Figure 2). Western blot analysis showed that eRF1iso2 was associated with all tested 60S ribosomal subunits (Figure 2). Interestingly, we found eRF1iso2 not only in the human ribosomes (HeLa and placenta) but also in the rabbit (rabbit reticulocyte lysate, RRL) and mouse (mouse Krebs-2 ascites lysate, Krebs-2) ribosomal subunits. At the same time, eRF1iso1 remains associated with the 40S subunits of various ribosomes, and its presence in 60S HeLa is likely explained by slight contamination of this particular preparation by 40S HeLa (Figure S2). Thus, we have shown that eRF1iso2 is expressed in human, rabbit, and mouse tissues and cell lines and could be detected bound to the large ribosomal subunits. Therefore, this form of eRF1 is rather conserved and obviously involved in translation.

### 2.2. eRF1iso2 Binds with the 80S Ribosome and with the Pre-Termination Complex

**Figure 3:**
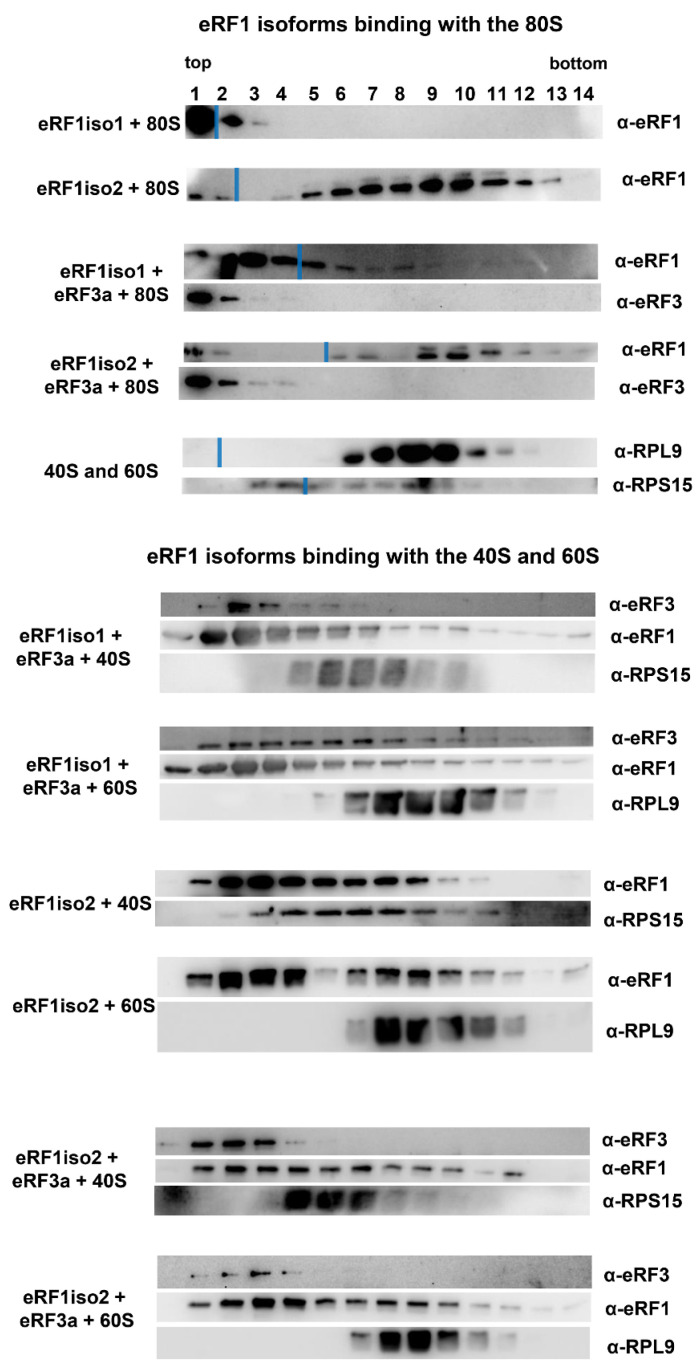
eRF1iso2 binding with purified 80S ribosomes and ribosomal subunits. Western blot analysis of 80S ribosomes resolved by sucrose density gradients and 40S and 60S ribosomal subunits after incubation with eRF1iso1 or eRF1iso2 in the presence and absence of eRF3a. Antibodies raised against eRF1, eRF3a, rpL9, rpS15 were used for detection. The fractions of the SDG are indicated above the Western blots; fraction 1 corresponds to the top of the gradient and fraction 14 to the bottom. Blue lines indicate the junction of Western blot images after Protein Marker lane cutting, which was performed to facilitate comparison of matched samples (raw data available). 80S ribosomes were assembled from 40S and 60S subunits purified from RRL.

In order to study how eRF1iso2 interacts with the ribosomes, we compared an ability of both eRF1 isoforms to bind 80S ribosomes and 40S and 60S ribosomal subunits in the presence or absence of eRF3a (Figure 3). Ribosomes or ribosomal subunits were incubated with eRF1 isoforms, eRF3a, and GTP, and then the complexes were centrifuged in a sucrose density gradient (SDG). After fractionation, proteins were detected by Western blotting. The 60S subunits migrated in fractions 6–11 and 40S in fractions 3–8 (Figure 3). To exclude the aggregation of proteins in solution, we determined their distribution in the SDG in the absence of ribosomes (Figure S3A). All proteins (eRF1iso1, eRF1iso2, and eRF3a) were detected only in the top fractions of the gradient (fractions 1–5). In the presence of the 80S ribosomes, only eRF1iso1 was detected only in the top fractions of the gradient (fractions 1–3); however, in the presence of eRF3a, a small amount of the eRF1 iso1 was detected in the middle part of the gradient, indicating minor binding to 80S. It is worth noting that the sensitivity of the anti-eRF3a antibodies used was lower than that of the anti-eRF1 antibodies, which is why inadequate amounts of eRF3a in the gradient may not be detected (Figure S3B).

On the contrary, eRF1iso2 was found in the fractions 4–13, both in the presence or absence of eRF3a, and consequently, it was bound to 80S (Figure 3). Interestingly, in the absence of eRF3a, eRF1iso2 was bound to the 80S more tightly than in the presence of this protein, as the amount of bound eRF1iso2 without eRF3a was significantly higher. Thus, the presence of eRF3a in the binding reactions slightly destabilized the binding of eRF1iso2 to the 80S. Moreover, in the presence of individual ribosomal subunits eRF1iso2 was found both in the 40S and 60S fractions. We also observed binding of eRF1iso1 with individual ribosomal subunits, which indicated the presence of binding sites on subunits that are not accessible in the 80S ribosome. It should be noted that the ribosomal subunits used from RRL exhibited minor cross-contamination, not exceeding 5%, which is inevitable with standard SDG separation techniques (Figure S2). It is this that explains the sometimes observed distributions of bound proteins downstream of ribosomal subunits in the gradient upon 40S binding. Summarizing the results obtained, we can conclude that eRF1iso2 binds more stably to 80S ribosomes than eRF1iso1, and eRF3a weakly suppresses this interaction.

**Figure 4:**
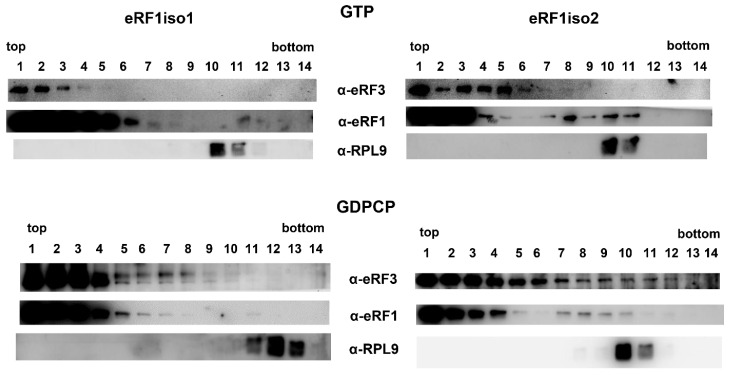
eRF1iso2 binding with the preTC. Western blot analysis of SDG of the preTC after incubation with eRF1iso1 or eRF1iso2 in the presence of eRF3a and GTP or GDPCP. Antibodies raised against eRF1, eRF3a, and rpL9 were used for detection. The fractions of the SDG are indicated above the Western blots; fraction 1 corresponds to the top of the gradient and fraction 14 to the bottom.

Then, we studied how eRF1iso2 interacts with the pre-termination (preTC) ribosomal complex, where the ribosomal A site contains a stop codon (Figure 4). We obtained the preTC in the reconstituted translation system and purified it from the translational factors by ultracentrifugation in a sucrose gradient. PreTC was incubated with eRF1iso1 or eRF1iso2, eRF3a, and GTP or GDPCP. Subsequently, the complexes were centrifuged in the SDG and analyzed by Western blotting. PreTC migrated in the fractions 10–13. eRF1iso1 was found only predominantly in the fractions 1–6 in the presence of both types of nucleotides, so we did not determine its stable interaction with the preTC, while barely detectable amounts of eRF1 were found with the preTC. eRF3a interacting with eRF1iso1 also did not stably bind with the preTC in the presence of GTP or GDPCP. In contrast, eRF1iso2 was found in the preTC fractions both in the presence of GTP and GDPCP. Moreover, in the presence of non-hydrolysable analog it facilitated the binding of eRF3a to the preTC. Therefore, we conclude that eRF1iso2 efficiently binds with the preTC and stabilizes the binding of eRF3a to the ribosomal complex before GTP hydrolysis.

It should be noted that the binding experiments were carried out in the absence of cross-linking agents and therefore only detect strong and stable interactions since when the ribosomal complexes are ultracentrifuged in a sucrose gradient without cross-linking the complexes with formaldehyde, unstable complexes dissociate but stable ones remain intact. Labile interactions are less easily detected by this method. This may explain the weaker binding of eRF1iso1, which normally should be constantly exchanged on the ribosome and is therefore less detectable after centrifugation in SDG. But, in order to compare the efficiency of the binding of the eRF1 isoforms to ribosomes, the preTC, and eRF3a, we did not detect cross-link interactions.

### 2.3. Reduced Ability of eRF1iso2 to Form a Translation Termination Complex

**Figure 5:**
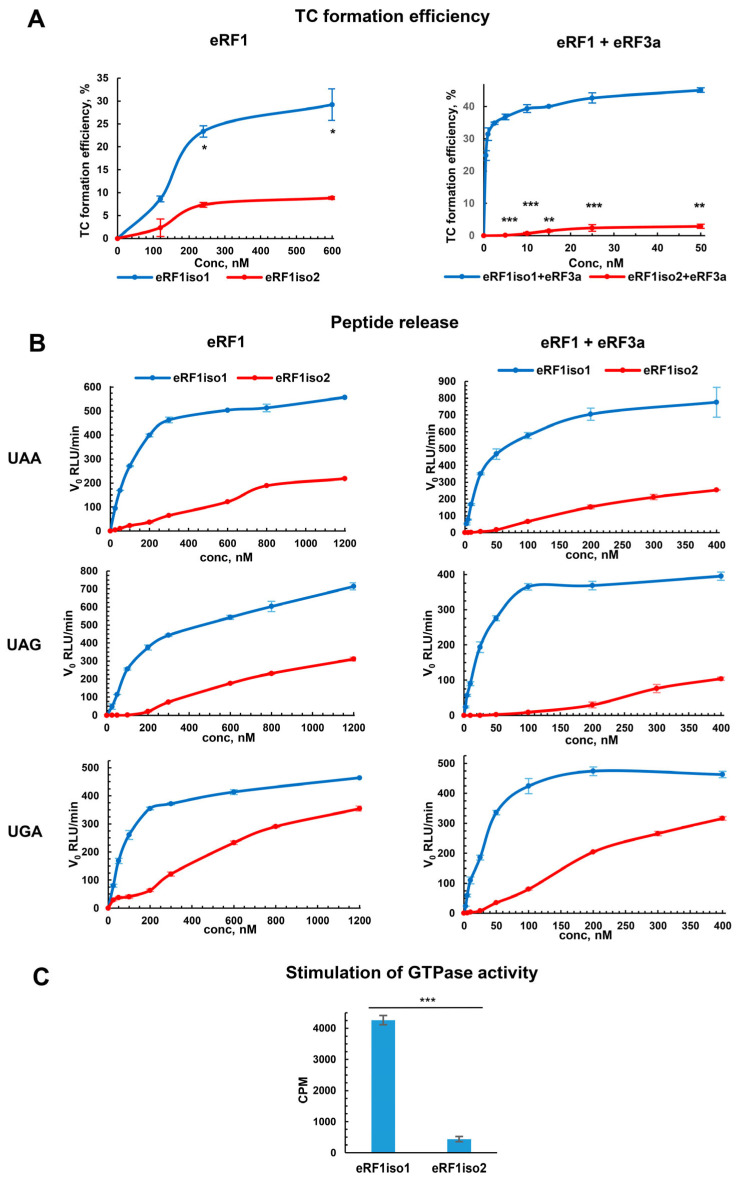
Functional activity of eRF1iso2 in translation termination. (**A**) Termination complex formation efficiency by eRF1iso1 and eRF1iso2 in the presence and absence of eRF3a. Toe-print analysis of TC formed at UAA stop codon in the various concentrations of eRF1 isoforms (n = 3). (**B**) Hydrolysis of peptidyl-tRNA induced by eRF1iso1, eRF1iso2, and eRF3a. Termiluc assay on the preTCs assembled at UAA, UAG, and UGA stop codons. v_0_ is the initial termination rate (RLU/min) (n = 3). (**C**) GTPase activity of eRF3a in the presence of eRF1iso1 and eRF1iso2 (n = 3). The error bars represent the standard deviation. Background activity measured in the presence of all components except eRF1 was subtracted. Asterisks indicate statistically significant differences (*, *p* < 0.05; **; *p* < 0.01; ***, *p* < 0.001).

The functional activity of eRF1iso2 was studied in all stages of translation termination in which eRF1 is involved: binding to the stop codon in the ribosome, induction of peptidyl-tRNA hydrolysis and stimulation of eRF3 activity during GTP hydrolysis. First of all, using fluorescent toe-printing analysis, we determined the ability of eRF1iso2 to bind the stop codon in the ribosome and form termination complexes in the reconstructed translation system [3]. Here, we used the preTCs purified from all the translational factors by ultracentrifugation in high-salt sucrose gradient. Fluorescent toe-printing is based on the reverse transcription reaction with fluorescently labeled primers annealing downstream of the ribosomal complex [27,48]. The position of the ribosome on mRNA is calculated via the length of the fragment, which extends the primer during reverse transcription. Toe-prints corresponding to the termination complexes (TCs) are located for 1–2 nucleotides downstream of the toe-prints corresponding to the preTCs, since during eRF1 binding the stop codon in mRNA adopts a U-turn structure and the ribosome protects additional nucleotides. It allows us to determine the TC formation efficiency. We observed that on the pure preTCs, eRF1iso2 formed TCs at the stop codon worse than the main form eRF1iso1; moreover, the difference between the two isoforms of eRF1 even increased in the presence of eRF3a (Figure 5A and Figures S3, S4 and S6). These data at first glance contradict the results of eRF1iso2 binding to the ribosomal complexes (Figure 4), since we have shown that eRF1iso2 is more strongly bound to the preTC than eRF1iso1. It could be assumed that due to the loss of the part of the N-domain with the GTS loop, important for stop codon recognition, eRF1iso2 is unable to form a U-turn structure, nevertheless remaining associated with the A site of the ribosome. Another explanation of this contradiction could be that eRF1iso2 has an additional binding site on the ribosome. To find out which of these two hypotheses is correct, we conducted experiments on the competition of eRF1iso2 with eRF1iso1 for binding to the stop codon. We observed that an excess of eRF1iso2 does not affect the efficiency of TC formation by the complex eRF1iso1–eRF3a (Figure S5). Thus, eRF1iso2 likely has an additional binding site in the preTC. Therefore, removal of the N-terminal 33-a.a. fragment reduces eRF1 affinity to the stop codons.

### 2.4. Decreased Ability of eRF1iso2 to Induce Peptide Release

The ability to induce peptidyl-tRNA hydrolysis by eRF1iso2 was determined using a Termi-luc assay [49]. In this assay, we used preTCs (purified from RRL) containing nanoluciferase (Nluc), the release of which from the ribosome during translation termination caused luminescence of the substrate. We observed that the peptidyl-hydrolysis activity of eRF1iso2 was significantly lower than that of eRF1iso1 at all stop codons (Figure 5B and Figure S6).

However, eRF1iso2 recognized the UGA codon better than two other codons. After the addition of eRF3a, the low activity of eRF1iso2 at all three stop codons was preserved (Figure 5B and Figure S6). Thus, a lack of the N-terminus reduces the ability of eRF1iso2 to hydrolyze peptidyl-tRNA. These data are consistent with our data on the termination complexes formation by eRF1iso2 (Figure 5A), where deletion of the N-terminus reduced its ability to bind to the stop codons.

Based on the dependence of the initial rate of peptidyl-tRNA hydrolysis on the concentration of release factors, we calculated the apparent Michaelis constant (K_M_), which characterizes the ability of the protein to bind to the ribosome, and the maximum reaction rate (v_max_), which characterizes the rate of peptidyl-tRNA hydrolysis (Figure 5B and Figure S6, Table 1). We found that the K_M_ of eRF1iso2 was approximately 20–40-fold higher than that of eRF1iso1 at all stop codons both in the presence and absence of eRF3a. The v_max_ values, on the contrary, differed slightly in the absence and presence of eRF3a. Thus, eRF1iso2 has reduced ability to bind to the stop codons in the ribosome, which does not affect the ability of the ribosome to hydrolyze peptidyl-tRNA.

### 2.5. eRF1iso2 Interacts with eRF3a in Solution but Is Unable to Induce Its GTPase Activity

**Figure 6:**
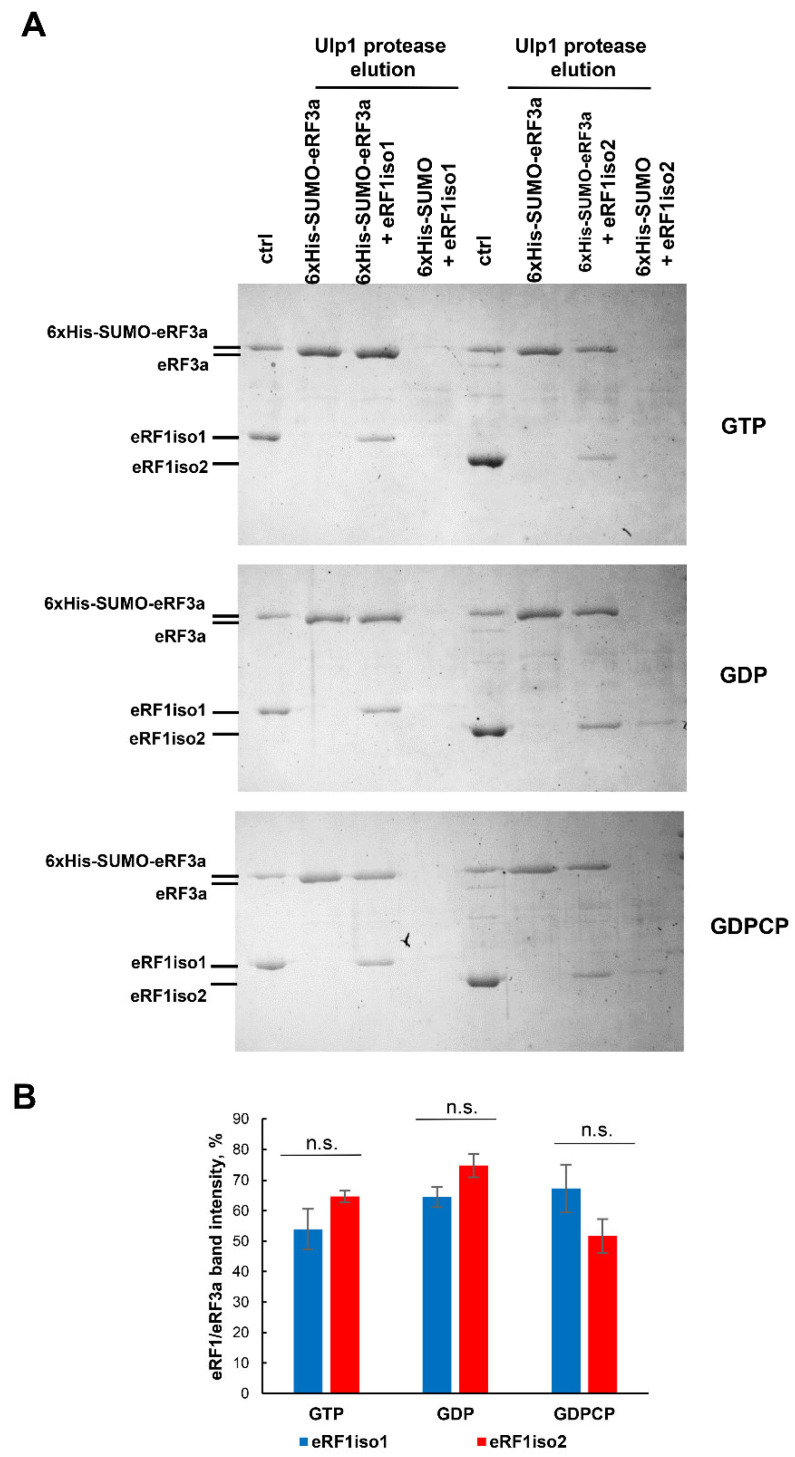
Binding of eRF1iso2 with eRF3a in solution. (**A**) Representative figure of the binding of eRF1iso1 and eRF1iso2 to eRF3a determined by pull-down of His-SUMO-eRF3a in the presence of eRF1 isoforms. Protein samples before loading onto the Ni-NTA resin (ctrl) or after cleavage by Ulp1 protease were analyzed by SDS electrophoresis. All experiments were carried out in three replicates. (**B**) The intensities of the eRF1 bands were normalized to the intensity of the eRF3a band. Histogram data are presented as mean relative intensity ± standard error of the mean. The difference was considered significant when *p* value (two-tailed *t*-test) was less than 0.05. n.s., non-significant difference (*p* ≥ 0.05).

Then we performed a GTPase activity induction test in the presence of two isoforms of eRF1. We have shown that eRF1iso2 induced the GTPase activity of eRF3 significantly worse than eRF1iso1 (Figure 5C). To find out whether eRF1iso2 was able to interact with eRF3a in solution, we studied the binding of these proteins in the presence of different nucleotides using a pull-down assay (Figure 6). For this purpose, 6xHis-SUMO-eRF3a was incubated with eRF1iso1 or eRF1iso2 in the presence of GTP, GDP, or GDPCP and then bound with Ni-NTA agarose. The bound eRF3–eRF1 protein complex was cut from the resin by Ulp1 protease and analyzed by denaturing electrophoresis. We found that in the presence of all tested nucleotides, eRF1iso2 binds to eRF3a in solution with the same efficiency as eRF1iso1 (Figure 6B). Thus, efficient binding of eRF1iso2 to eRF3a nevertheless practically does not activate its enzymatic activity.

Summarizing all obtained data on the activity of eRF1iso2 in translation termination, we can conclude that it binds to stop codons weaker than eRF1iso1 and almost does not stimulate the GTPase activity of eRF3, which results in a decrease in its ability to induce peptidyl-tRNA hydrolysis, both in the absence and in the presence of eRF3a.

### 2.6. Effect of eRF1iso2 on the Stop Codon Readthrough and Translation

**Figure 7:**
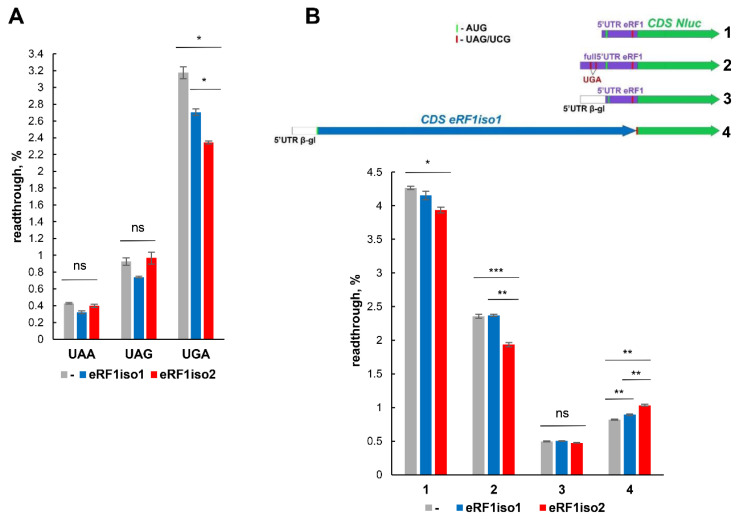
Effect of eRF1iso2 on the stop codon readthrough. (**A**) Effect of excess of eRF1 isoforms on the stop codon readthrough in HEK293 lysate on Fluc reporter mRNAs with different PTCs. (**B**) Effect of excess of eRF1 isoforms on the stop codon readthrough in HeLa lysate on the Nluc reporter mRNAs with different leader sequences. All experiments were carried out in three replicates. Histogram data are presented as mean relative intensity (readthrough, %) ± standard error of the mean. The difference was considered significant when *p* value (two-tailed *t*-test) was less than 0.05 (*). n.s., non-significant difference (*p* ≥ 0.05). Asterisks indicate statistically significant differences (*, *p* < 0.05; **; *p* < 0.01; ***, *p* < 0.001).

Since eRF1iso1 may suppress readthrough of premature stop codons (PTCs) via stimulation of translation termination, we decided to investigate how eRF1iso2 affects readthrough. To this purpose, mRNA encoding firefly luciferase (Fluc) and carrying three different premature stop codons at position Y447 (Figure 7A) was translated in a cell-free translation system, obtained from HEK293 cells, in the presence of eRF1iso1 or eRF1iso2. The level of readthrough was determined by the ratio of Fluc-stop/Fluc-wt translation. It turned out that while eRF1iso1 weakly reduced the level of readthrough of all three stop codons, eRF1iso2 reduced the level only of UGA readthrough but more strongly than isoform 1 (Figure 7A). This selectivity of the UGA stop codon correlates with an increased level of peptidyl-tRNA hydrolysis at UGA codon compared to UAA and UAG (Figure 5B). It should be noted that in all the lysates we studied, there was a large amount of endogenous eRF1, reaching, according to our estimates, a concentration of 0.1–0.2 µM and represented predominantly by isoform 1 [50]. The additional amounts of recombinant eRF1 isoforms we added were comparable to the amount of endogenous eRF1. Under such conditions, the differences in the level of readthrough we observed are statistically significant.

In addition, we performed experiments to determine the level of readthrough in another model system using mRNA encoding Nluc which was preceded by a leader sequence and the beginning of the β-globin CDS. Between β-globin and Nluc coding sequences, three stop codons were inserted in different “weak” 3’ contexts [51] (Figure S7). As a control, mRNAs were used in which stop codons were replaced by near-cognate sense codons. In this system, PTC is located closer to the 5’ end of the mRNA, which could serve as a model of the uORF readthrough. In the cell-free translation system produced from HeLa lysate, we found that readthrough at the UAA and UAG stop codons was independent of either eRF1iso1 or eRF1iso2 (Figure S7). However, at the UGA stop codon, eRF1iso1 reduced readthrough, while eRF1iso2 increased it. Thus, the readthrough of stop codons located in different regions of mRNA is regulated differently by two eRF1 isoforms.

To evaluate the possibility of eRF1 translation regulation by the isoforms, we determined the level of stop codon readthrough on the mRNA coding the main eRF1 isoform. For this purpose, we obtained four types of mRNA (Figure 7B). All of them contained Nluc and the 3′UTR of eRF1 and different leader sequences: (1) truncated 5′UTR of the eRF1 mRNA; (2) full-length 5′UTR of the eRF1 mRNA; (3) 5′UTR of β-globin mRNA and subsequent truncated 5′UTR of the eRF1 mRNA; and (4) 5’UTR of β-globin mRNA and CDS of eRF1. All of these mRNAs were constructed in pairs with the UAG stop codon before Nluc and with the UCG codon. This allowed us to determine the level of readthrough of the stop codon based on the ratio of signals from translation at stop and sense codons. In the cell-free translation system obtained from HEK293 lysate, we found that on mRNA carrying a shortened 5’UTR of eRF1 mRNA, as well as on the full-length 5’UTR of eRF1, eRF1iso2 stop codon readthrough decreased; however, on mRNA with eRF1 CDS, stop codon readthrough even increased (Figure 7B). Interestingly, the reduction in readthrough in the presence of eRF1iso2 is greater on mRNAs with the full-length 5’UTR of eRF1, containing additional UGA stop codons in uORFs. Obviously, the surrounding nucleotide sequence and the proximity of stop codons to the 5’ or 3’ ends of the mRNA play a significant role in the effect of eRF1iso2 on stop codon readthrough.

**Figure 8:**
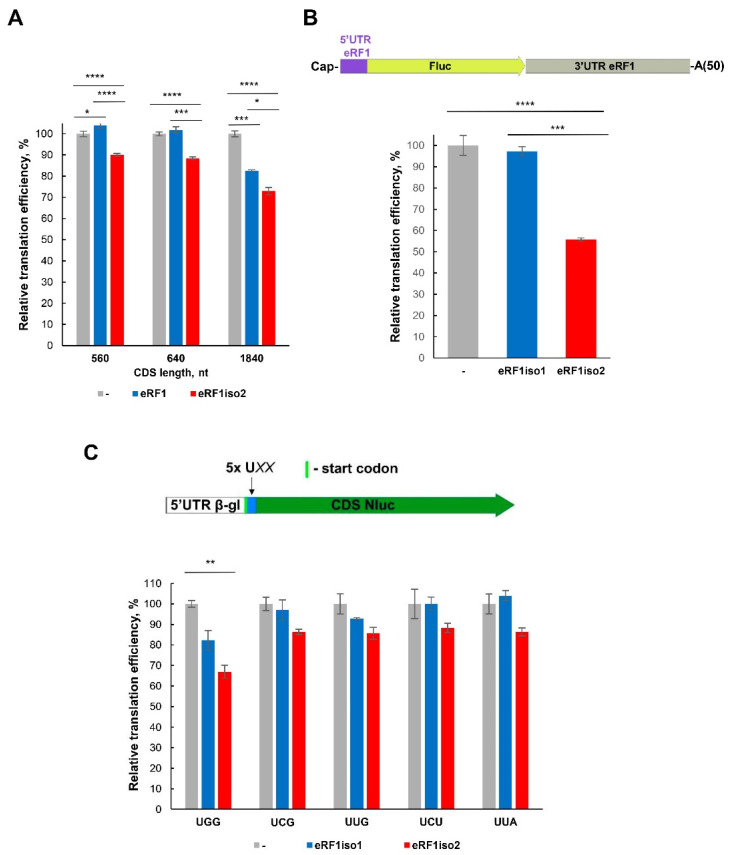
Effect of eRF1 isoforms on translation. (**A**) Translation of Nluc mRNAs with various CDS lengths in the presence of eRF1 isoforms in HeLa lysate. (**B**) Translation of Fluc mRNA in the presence of eRF1 isoforms in HEK293 lysate. (**C**) Translation of Nluc mRNAs with the additional sense codons in the presence of eRF1 isoforms in HEK293 lysate. 5x UXX–repeat of 5 identical codons, each of UGG, UCG, UUG, UCU, or UUA. All experiments were carried out in three replicates. Histogram data are presented as mean relative intensity (relative translation efficiency, %) ± standard error of the mean. The difference was considered significant when *p* value (two-tailed *t*-test) was less than 0.05 (*). Asterisks indicate statistically significant differences (*, *p* < 0.05; **; *p* < 0.01; ***, *p* < 0.001; ****, *p* < 0.0001).

To study the effect of eRF1iso2 on translation, we performed translation of mRNA with different lengths of coding sequences in the cell-free systems based on HeLa and HEK293 lysates (Figure 8A,B and Figure S8). We found that eRF1iso2 inhibited translation in direct ratio to the length of the translated sequence (Figure 8A,B and Figure S8). At the same time, eRF1iso1 slightly suppressed translation only at the longest CDS. We supposed that this inhibition of translation by eRF1iso2 was caused by the competition of eRF1iso2 with aminoacyl-tRNA for binding to the ribosome during elongation.

Additionally, we checked whether there is a dependence of translation inhibition by eRF1iso2 on the codon composition of the translated template. Since eRF1iso2 retains the NIKS motif responsible for recognition of U in the first position of the stop codon, we selected for such testing several sense codons with U in the first position. Among these codons, we chose mainly those rarely found in humans, expecting an increased probability of their suppression by eRF1iso2. We constructed nanoluciferase mRNAs with repeats of five identical codons after the start codon in the main reading frame (Figure 8C). The control for such constructs is Nluc mRNA with a long CDS of 560 nt (Figure 8A). The introduction of additional codons did not significantly affect luciferase activity, and the effect of eRF1 isoforms on translation was compared for each mRNA separately. eRF1iso2, unlike isoform 1, additionally suppressed translation at all stop codons studied, but this effect was not significantly different from that of the control Nluc 560 nt mRNA (Figure 8A), with the exception of UGG. By increasing the number of UGG codons in the CDS, the addition of eRF1iso2 resulted in a significantly greater reduction in translation (Figure 8C and Figure S8). At the same time, inhibition of translation on such a matrix was also observed with the addition of eRF1iso1 but to a much lesser extent. It is worth noting that UGG is most similar to stop codons and it is known that eRF1iso1 can bind to this codon [52,53]. Thus, our data show that eRF1iso2 discriminates UGG from stop codons even worse than the main isoform, due to which it is able to further inhibit translation (Figure 8A).

### 2.7. PABP Stimulates Translation Termination Induced by eRF1iso2

**Figure 9:**
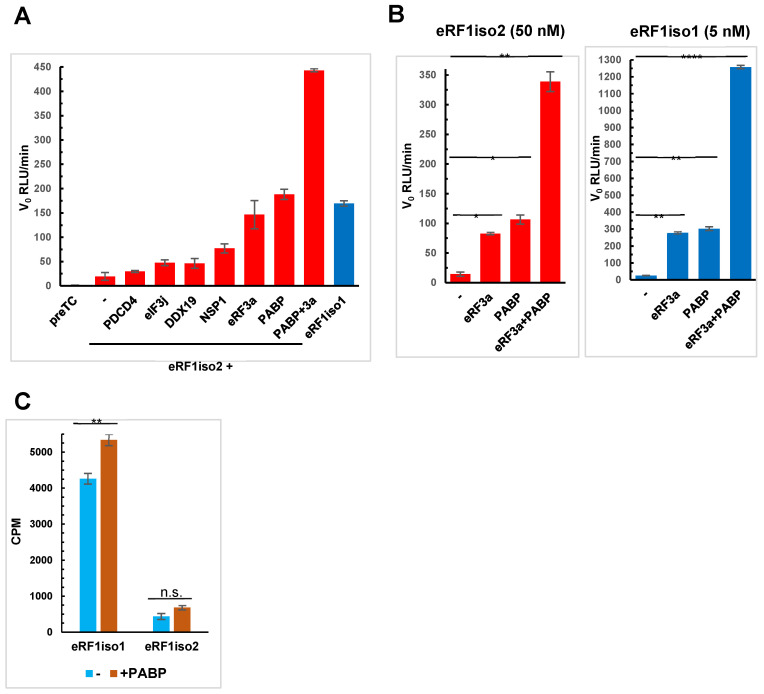
Effect of additional factors on translation termination induced by eRF1iso2. (**A**) Hydrolysis of peptidyl-tRNA induced by eRF1iso2 in the presence of PDCD4, eIF3j, DDX19, Nsp1, eRF3a, and PABP. Termiluc assay on the preTCs assembled at UAA stop codon. v_0_ is the initial termination rate (RLU/min) (n = 3). The red color corresponds to eRF1iso2 data, and the blue color corresponds to eRF1iso1 data. (**B**) Hydrolysis of peptidyl-tRNA induced by eRF1iso1 and eRF1iso2 in the presence of PABP and eRF3a. Termi-luc assay on the preTCs assembled at UAA (n = 3). The red color corresponds to eRF1iso2 data, and the blue color corresponds to eRF1iso1 data. (**C**) GTPase activity of eRF3a in the presence of eRF1iso1, eRF1iso2, and PABP (n = 3). Background activity measured in the presence of all components except eRF1 was subtracted. The error bars represent the standard deviation. n.s., non-significant difference (*p* ≥ 0.05). Asterisks indicate statistically significant differences (*, *p* < 0.05; **; *p* < 0.01; ****, *p* < 0.0001).

As the activity of eRF1iso2 in translation termination is significantly reduced relative to eRF1iso1, we decided to test the ability of some other factors to stimulate the eRF1iso2 activity in translation termination. Previously, it was shown that translation termination by eRF1iso1 is stimulated by several factors: PABP [30], DDX19 [38], PDCD4 [54], eIF3j [42], and NSP1 of SARS-CoV-2 [55]. We tested whether these factors stimulated eRF1iso2 activity and found that only PABP retained its stimulatory effect on translation termination induced by eRF1iso2, especially in the presence of eRF3a (Figure 9A and Figure S9). However, PABP stimulated the eRF1iso1 more strongly than eRF1iso2 (Figure 9B and Figure S9). In the presence of PABP, there is also a slight stimulation of the GTPase activity of eRF3a by eRF1iso2, which is still significantly lower than that of the main isoform (Figure 9C). Therefore, termination of translation induced by eRF2iso2 is not stimulated by additional tested proteins, except PABP, probably due to the lacking of the important for this stimulation part of eRF1. PABP facilitates the loading of eRF3a to the ribosome, so its effect on termination does not depend on the sequence of the N domain of eRF1.

## 3. Discussion

In this study, we estimated the role of isoform 2 of human eRF1 in translation. Formed as a result of alternative splicing, eRF1iso2 is shorter than eRF1iso1 by 33 amino acids from the N-terminus (Figure 1A). eRF1iso1 is encoded by the most abundant ETF1 transcript. At the same time, eRF1iso2 is encoded by at least four additional ETF1 transcripts that arise from alternative splicing and can be translated (Figure 1B). We showed that in cells, eRF1iso2 is bound to ribosomal subunits (Figure 2). eRF1iso2 recognizes stop codons weaker than eRF1iso1 and almost does not stimulate the GTPase activity of eRF3, which reduces its ability to induce peptidyl-tRNA hydrolysis (Figure 5). However, isoform 2 of eRF1 binds much more efficiently to the preTC and individual ribosomes than eRF1iso1 (Figure 3 and Figure 4). Translation regulation experiments showed that eRF1iso2 differently affects stop codon readthrough and suppresses translation (Figure 7 and Figure 8). Thus, the truncated form of eRF1 has lower termination activity than canonical eRF1 but may possibly be involved in the regulation of protein biosynthesis though binding to the ribosomal complexes.

Although eRF1iso2 binds to the ribosomes much better than eRF1iso1, it does not compete with canonical eRF1 in translation termination (Figure S5). Therefore, it probably binds to an additional site in the ribosome. We hypothesize that deletion of the first 33 amino acids (α1 helix) changes eRF1 conformation on the ribosome, possibly opening access to the ribosome-binding region in eRF1. Indeed, recombinant eRF1iso2 was unstable during expression and purification from *E. coli*. While eRF1iso1 was easily purified after expression in *E. coli*, eRF1iso2 precipitated under these conditions and could only be obtained using an additional SUMO tag. However, eRF1iso2 is found on eukaryotic ribosomes and binds to the 60S subunits; at the same time, eRF1iso1 remained associated predominantly with 40S subunits (Figure 2). Highly specific binding to ribosomes stabilizes eRF1iso2 in cells and increases its local concentration for participation in translation, even despite its low presence in the cell. Thus, the absence of the α1 helix in eRF1 could open additional determinants for binding to the ribosome.

We showed that eRF1iso2 also was able to bind to eRF3 (Figure 6) but almost did not stimulate its GTPase activity (Figure 5). Binding to eRF3a and stabilizing it in an inactive form may lead to a decrease in eRF3 concentration in the cytoplasm. If we take into account the efficient binding of eRF1iso2 to the ribosomes both in the absence and presence of eRF3a (Figure 3 and Figure 4), we can assume that isoform 2 may serve as a depot of eRF3 on the ribosome. This pathway of translation regulation may be useful for translation termination at remote from the poly(A) tail stop codons, for example, in the uORFs. It was previously shown that eRF3 is loaded into the ribosome by PABP [30]. It has been proposed that PABP also acts as a depot for release factors. In a study of the activity of the translation factor eIF3j, it was supposed that eukaryotic translation initiation factor eIF3 could be such a depot in the case of uORF stop codons [42]. The eIF3j–eRF3a–GTP complex can bind to eIF3 on the 40S subunit additionally positioning release factors near stop codons of uORFs to induce translation termination. eRF1iso2 may also serve as an additional depot for eRF3a during translation termination at the uORFs. This assumption is supported by our data on the effect of eRF1iso2 on the stop codon readthrough in uORFs (Figure 7B). On two mRNAs containing uORFs, eRF1iso2 was able to reduce readthrough, indicating stimulation of translation termination.

eRF1iso2 differs from eRF1iso1 only in the absence of the first 33 amino acids, and, therefore, the study of this isoform further provides insight into the role of these missing amino acids for the functioning of the main eRF1 isoform. Our experiments showed that despite the reduced ability to hydrolyze peptidyl-tRNA, eRF1iso2 still recognized UGA stop codon better than the two others (Figure 5B, Table 1). Thus, our data are in good agreement with the assumption that the GTS loop forms a switch that is key for the multiple codon recognition capability of eRF1 [21,56]. Indeed, it was previously shown that the T32 mutation in the GTS loop of eRF1 directly leads to UGA unipotency of eRF1 [8,12]. These data are supported by structural studies that demonstrate that T32 of eRF1 interacts with purines at position 3 in the UAG [14] and UAA [15] but not UGA. It has also been shown that other eRF1 mutations that cause UGA unipotency act indirectly through changes in the GTS loop conformation, without changing the global conformation of the eRF1 N-domain [21,56]. It should be noted that eRF1iso2 recognizes UGA better than UAG and UAA but significantly worse than eRF1iso1 (Figure 5, Table 1). This is probably due to the absence of S33 in eRF1iso2 from the GTS loop since it is known that mutation of this amino acid leads to a decrease in the ability of eRF1 to recognize the UGA stop codon [8,9]. Thus, the effects of disruption of the GTS loop conformation correlate with the activity we observed in eRF1iso2 lacking this motif.

Our experiments also show that eRF1iso2 has a disrupted functional, but not physical, interaction with eRF3a. It is obvious that the determinant of such uncoupling is most likely located within the first 33 amino acids of eRF1iso1. In previous work by Wong et al., it has been shown that the helix α1 of N-domain eRF1 can interact with the decoding region of Helix 44 of 18S rRNA in the 40S subunit. A structural model of this interaction has been proposed, suggesting its importance in the conformational rearrangements of eRF1 in the A-site of the ribosome that occur after hydrolysis of GTP by eRF3, which ensure the hydrolysis of peptidyl-tRNA at the P-site of the ribosome [21]. Our data are consistent with this model since eRF1iso2, lacking the helix α1 of the N-domain, is not able to effectively stimulate the GTPase activity of eRF3a and thus induce peptide release. The obtained data on the activity of eRF1iso2 are in close agreement with the data of an early study of the N-terminal deletion mutants of eRF1iso1, for which reduced termination activity and uncoupling of functional activity from eRF3a were also observed [17]. However, it should be noted that, N-truncated forms of eRF1 with a 16-a.a. extension, including the 6xHis tag at the N-terminus were used in that study. Thus, eRF1iso2 is less active in translation termination, exhibiting greater affinity for UGA and is able to selectively inhibit translation in a manner dependent on the length of the CDS and its codon composition. Such properties may be useful in the cell to regulate translation when the concentration of eRF1iso1 is decreased, for example, during stress or cell specialization. Summarizing all the obtained results, we can assume that eRF1iso2 could be involved in regulation of translation and termination under certain conditions, which remains to be clarified in further studies. Additionally, our data allow us to evaluate the functional role of the first 33 amino acids of eRF1.

## 4. Materials and Methods

### 4.1. Plasmids and Model mRNAs

Plasmids for protein expression and mRNA are described in Supplementary Materials (Tables S1 and S2). The mRNA used in this work was obtained from PCR products containing the T7 promoter via run-off transcription with the T7 RiboMAX™ Large Scale RNA Production System (Promega, Madison, WI, USA) kit according to the manufacturer’s protocol. Templates and primers for obtaining the corresponding PCR products are given in Table S3. The construction of mRNA encoding MVHL with the different stop codons and the standard context was described previously [51]. To obtain capped mRNA, the transcription reaction mixture was additionally supplied with 3 mM 3’-O-Me-m7G(5’)ppp(5’)G RNA Cap Structure Analog (ARCA, NEB, Ipswich, MA, USA) in a 3-fold excess relative to GTP. All used mRNAs contained a 50 nt poly(A) tail.

### 4.2. Expression and Purification of Proteins

His-SUMO-tagged eRF1iso1, eRF1iso2, and eRF3a were expressed in *E. coli* BL21(DE3) (Invitrogen, Waltham, MA, USA) after induction by 0.5 mM isopropyl β-D-1-thiogalactopyranoside at 18 °C overnight, followed by purification using a Ni-NTA gravity flow column and elution by elution buffer (EB) containing 20 mM Tris-HCl pH 7.5, 500 mM KCl, 7.5% glycerol, 300 mM imidazole, and 1 mM DTT. Then, proteins were dialyzed against cleavage buffer (CB), comprising 20 mM Tris-HCl (pH 7.5), 250 mM KCl, 7.5% glycerol, 1 mM DTT. His-SUMO tag was cleaved by His-tagged peptidase Ulp1 in CB overnight at +4 °C and then purified with Ni-NTA agarose using gravity flow column. The product was dialyzed in buffer containing 20 mM Tris-HCl (pH 7.5), 100 mM KCl, 7.5% glycerol, 1 mM DTT. eRF1iso1, eRF1iso2, and eRF3a were then applied to a HiTrapQ column (GE Healthcare, Chicago, IL, USA) and purified by 100–500 mM gradient of KCl. The products were dialyzed in a storage buffer (SB) containing 20 mM Tris-HCl (pH 7.5), 100 mM KCl, 7.5% glycerol, 1 mM DTT.

40S and 60S ribosomal subunits and translation factors eIF2, eIF3, eEF1, eEF2 were purified from the RRL or HeLa cell lysates as described previously [3]. Other human translation factors were produced as recombinant proteins in the *E. coli* BL21(DE3) or Rosetta(DE3) (eIF1, eIF1A, eIF4A, eIF4B, ΔeIF4G, ΔeIF5B, eIF5) or in the insect cell line Sf21 with baculovirus EMBacY from a MultiBac expression system (eRF3a). Then, recombinant proteins were purified using Ni-NTA agarose and ion-exchange chromatography, as described previously [3,30].

### 4.3. Western Blot Analysis of Ribosomes

40S and 60S ribosomal subunits, as well as control samples of eRF1iso1 and eRF1iso2, were mixed with an equal volume of 2x Laemmli sample buffer and heated at 95 °C for 2 min. Afterward, the samples were separated by Glycine–SDS-PAGE (10%). Transferring was performed using the Trans-Blot Turbo system (Biorad, Hercules, CA, USA). The presence of eRF1 isoforms was detected by immunostaining with the eRF1 antibodies (see Supplementary Materials).

### 4.4. Pre-Termination Complex Assembly and Toe-Printing

Pre-termination complexes on MVHL-stop mRNA in the eukaryotic reconstituted translation system were assembled and purified as previously described [3]. Briefly, initiation complexes were assembled in a 500 µL solution containing 37 pmol MVHL-stop mRNA, 200 pmol Met-tRNA_i_^Met^, 90 pmol 40S and 60S ribosomal subunits, 200 pmol eIF2, 90 pmol eIF3, and 125 pmol of eIF4A, ΔeIF4G, eIF4B, eIF1, eIF1A, eIF5, ΔeIF5B, respectively, supplemented with buffer A, containing 25 mM Tris-HCl (pH 7.5), 50 mM KOAc, 2.5 mM MgCl_2_, 2 mM DTT, 0.3 U/µL RNase inhibitor, 1 mM ATP, 0.25 mM spermidine, and 0.2 mM GTP. The reaction mixture was incubated at 37 °C for 15 min to allow ribosomal-mRNA complex formation. Peptide elongation was performed by adding 200 pmol total tRNA (acylated with all or individual amino acids), 200 pmol eEF1H, and 50 pmol eEF2 to the initiation complex, followed by incubation for another 15 min at 37 °C. The ribosomal complexes were centrifuged in a Beckman SW55 rotor for 95 min at 4 °C and 50000 rpm in a linear sucrose density gradient (10–30%, *w*/*w*) prepared in buffer A containing 5 mM MgCl_2_. The preTC fractions were combined and diluted to a final concentration of 2.5 mM Mg^2+^ and used in toe-printing analysis and preTC binding assay.

Fluorescent toe-print analysis was performed as described [27,48]. The corresponding quantities of iso1/iso2 or iso1/iso2 with 0,05 pmol eRF3a (supplemented with 0.2 mM GTP and 0.2 mM MgCl_2_) were added to 8 μL of the purified preTC (MVHL-UAA) in SB to the final volume of 10 μL. The reaction was incubated for 15 min at 37 °C with the following incubation for 1 h at 37 °C with AMV reverse transcriptase (NEB, Ipswich, MA, USA) and a 5′-FAM-labeled primer. Obtained cDNA was purified and analyzed on the ABI Prism^®^ Genetic Analyser 3100 (Applera, Norwalk, CA, USA) or the Syntol LLC Nanophor 05 Genetic Analyser (Syntol, LLC, Moscow, Russia).

### 4.5. Ribosome and preTC Binding Assay

Individual ribosomal subunits or their mixture (15 pmol each) or purified preTCs (100 μL) were incubated with 10 pmol eRF1 (iso1 or iso2) and 10 pmol eRF3a (if necessary) in buffer A with 0.2 mM GTP (or GDPCP) and 0.2 mM MgCl_2_ at 37 °C for 10 min. The corresponding reaction mixture in a volume of 200 μL was purified on a linear sucrose density gradient (SDG) of 10–30% (wt) as described above. The gradients were fractionated into 14 equal fractions, followed by precipitation in 10% trichloroacetic acid. Protein pellets were analyzed by Western blotting.

### 4.6. Purification of the preTC-Nluc and Termi-luc Assay

Pretermination ribosomal complexes, translating Nluc (preTC-Nluc), for Termi-luc assay were purified as described [49,55]. 100% RRL lysate (Green Hectares, Thousand Oaks, CA, USA) was pre-incubated in a mixture containing 1 mM CaCl_2_ and 3 U/µL Micrococcal nuclease (ThermoFisher, Waltham, MA, USA) at 30 °C for 10 min, followed by the addition of EGTA to a final concentration of 4 mM. Then, lysate was diluted to 70% (*v*/*v*) and supplemented with 20 mM HEPES-KOH (pH 7.5), 80 mM KOAc, 0.5 mM Mg(OAc)_2_, 0.3 mM ATP, 0.2 mM GTP, 0.04 mM each of 20 amino acids (Promega, Madison, WI, USA), 0.5 mM spermidine, 0.45 µM aminoacyated total rabbit tRNA, 10 mM creatine phosphate, 0.003 U/µL creatine kinase (Sigma Aldrich, Burlington, NJ, USA), 2 mM DTT, and 0.2 U/µL Ribolock (Thermo Fisher, Waltham, MA, USA) (70% RRL mix).

For the preTC-Nluc assembly, 220 µL of 70% RRL mix was pre-incubated in the presence of 1.7 μM eRF1 mutant protein G183A (eRF1(AGQ)) at 30 °C for 10 min, followed by the addition of 10.5 pmol of NLuc mRNA. The mixture was incubated at 30 °C for 40 min. Next, KOAc concentration was adjusted to 300 mM and the mixture was layered on 5 mL of 10–35% linear sucrose gradient in buffer containing 50 mM HEPES-KOH, pH 7.5, 7.5 mM Mg(OAc)_2_, 300 mM KOAc, 2 mM DTT. The gradient was centrifuged in a SW55-Ti (Beckman Coulter, Brea, CA, USA) rotor at 55,000 rpm (367,598 gmax) for 1 h. The gradient was fractionated by 150 μL from bottom to top into 15 fractions. Fractions were analyzed in Termi-luc assay and stored at −70 °C.

Termi-luc assay was performed as previously described with modifications [49]. Peptide release was performed in a solution finally containing 1.5 pM preTC-Nluc, 45 mM HEPES-KOH pH 7.5, 1.4 mM Mg(OAc)_2_, 56 mM KOAc pH 7.0, 1 mM DTT, 177 µM spermidine, 1.5% (*w*/*w*) sucrose, 0.8 mM MgCl_2_, 0.2 mM GTP supplemented with equimolar MgCl_2_, 0.5% NanoGlo (Promega, Madison, WI, USA) in the presence of release factors in various concentrations. Luminescence was measured at 30 °C using a Tecan Infinite 200 Pro (Tecan, Männedorf, Switzerland). The translation termination efficiency was calculated as a maximal derivative of the growing linear section of the luminescence curve (v_0_, RLU/min) with the subtraction of v_0_ in the absence of eRF1. v_0_ for various concentrations of release factors were used to calculate K_M_ and v_max_ by the non-linear approximation to the Michaeles–Menton function using the dir.MM function from the renz library for R [57].

### 4.7. GTPase Assay

The ribosomal 40S and 60S subunits (1 pmol each) were associated at 4 °C for 10 min, and then, a GTPase buffer (10 mM pH 8.0 Tris-HCl, 12 mM NH_4_Cl, 30 mM KCl, 6 mM MgCl_2_) containing 2 μM [^γ−32^P] GTP with a specific activity of 2.16 μCi/pmol was added. Next, 2 pmol of each protein was added to the reaction. The reaction was initiated by the addition of 2 pmol of eRF3a, and the final reaction volume was 10 μL. After incubation for 20 min at 30 °C, the reaction was stopped by the addition of 500 μL of a solution containing 5% charcoal in 50 mM NaH_2_PO_4_ to bind free GTP. Finally, the charcoal was precipitated via centrifugation at 12,000× *g*, and 380 μL of the supernatant was subjected to liquid scintillation counting to quantify the released [^32^P] phosphate.

### 4.8. Pull-Down Analysis

To study protein–protein interactions, an in vitro pull-down assay was performed using purified proteins as described [42,58] with modifications. His-Select^®^ Nickel Affinity Gel (Sigma-Aldrich, Burlington, NJ, USA) was equilibrated by wash buffer (WB) (20 mM Tris–HCl pH 7.5, 150 mM KCl, 5% (*v*/*v*) glycerol, 0.05% (*v*/*v*) Tween-20, 10 mM imidazole pH 7.5, 1 mM DTT). His-SUMO-tagged proteins were added to 25% resin in WB and mixed for 5 min, and then the resin was washed four times with a large excess of WB. 5 μL of 20% WB resin bound with SUMO-tagged protein was incubated for 30 min with 20 μL of proteins of interest at a concentration of 1 μM each in binding buffer (BB) (20 mM Tris–HCl pH 7.5, 50 mM KOAc, 75 mM KCl, 2.7 mM MgCl_2_, 5% (*v*/*v*) glycerol, 0.25 mM Spermidine, 2 mM DTT, and 0.2 mM nucleoside triphosphate (GTP, GDP, or GDPCP)). Then, the resin was washed four times with a large excess of WB. After addition of 20 µL cleavage buffer 1 (CB1) (20 mM Tris–HCl pH 7.5, 240 mM KCl, 5% (*v*/*v*) glycerol, 0.035% (*v*/*v*) Tween-20, 7 mM imidazole pH 7.5, 1 mM DTT) bound proteins were eluted by 0.1 µM Ulp1 cleavage during incubation for 40–60 min. Eluted proteins were analyzed in PAAG with Coomassie staining or Western blot hybridization.

### 4.9. In Vitro Translation in HEK293 or HeLa Lysate

HEK293T cells were cultivated in DMEM supplemented with 10% FBS and were grown at 37 °C and 5% CO2. HEK293 lysate was prepared as described [59,60,61,62,63]. HeLa lysate was from HeLa Cytoplasmic Extracts, Ipracell (CC-01-40-50) (Ipracell, Mons, Belgium). Translation of reporter mRNA was carried out in 10 μL of a mixture containing 50% (*v*/*v*) HEK293 (or HeLa) lysate, 20 mM HEPES-KOH pH 7.5, 2 mM DTT, 0.25 mM spermidine, 0.6 mM Mg(OAc)_2_, 16 mM creatine phosphate, 0.06 U/µL creatine kinase (SigmaAldrich, Burlington, VT, USA), 1 mM ATP, 0.6 mM GTP, 60 mM KOAc, 0.05 mM of each amino acid (Promega, Madison, WI, USA), 0.2 U/µL RiboLock (Thermo Scientific, Waltham, MA, USA), 5 mM D-Luciferin (or 1% Nano-Glo^®^ Luciferase Assay Substrate (Promega, Madison, WI, USA)). Release factors were added to a final concentration of 0.5 µM. The final concentrations of mRNAs with the Fluc reporter were 40 nM. The final concentrations of mRNAs with the Nluc reporter (Table S3) were selected by the maximum luminescence signal within the linear detection range. Luminescence was measured at 30 °C using a Tecan Infinite 200 Pro (Tecan, Männedorf, Switzerland) for 120 min. The translation efficiency was calculated as a maximal derivative of the growing linear section of the luminescence curve (v0, RLU/min). Stop codon readthrough was defined as the ratio of the translation efficiency of a template with the PTC to the corresponding template without the PTC, expressed in %.

### 4.10. Statistical Analysis

All experiments were carried out in at least 3 replicates. All data are presented as mean ± standard error of mean (SE). An unpaired two-tailed t-test was used to compare mean values between two groups. For multiple comparisons, the Holm correction of *p* value was used [64]. Asterisks indicate statistically significant differences (*, *p* < 0.05; **; *p* < 0.01; ***, *p* < 0.001; ****, *p* < 0.0001). *p* values were calculated using T.TEST function in Microsoft Excel or t_test function (rstatix library) in R. Adjusting was performed by p.adjust function (stats library) in R.

## Figures and Tables

**Table 1:** Kinetic parameters of translation termination at different stop codons depending on eRF1 isoforms.

Stop Codon	Release Factors	Parameter	eRF1iso1	eRF1iso2	Fold Change iso2/iso1	*p* Value
UAA	eRF1	K_M_, nM	118 ± 5	5194 ± 215	44	0.027 (*)
		v_max_, RLU/min	607 ± 27	1265 ± 24	2.1	0.0005 (***)
	eRF1+eRF3	K_M_, nM	40 ± 4	1089 ± 1	27	7.8 × 10^−6^ (****)
		v_max_, RLU/min	841 ± 64	965 ± 35	1.1	0.192 (n.s.)
UAG	eRF1	K_M_, nM	267 ± 14	10,119 ± 5192	38	0.309 (n.s.)
		v_max_, RLU/min	832 ± 11	3035 ± 1300	3.6	0.339 (n.s.)
	eRF1+eRF3	K_M_, nM	32 ± 3	nd	-	nd
		v_max_, RLU/min	440 ± 5	nd	-	nd
UGA	eRF1	K_M_, nM	95 ± 7	2149 ± 259	23	0.015 (*)
		v_max_, RLU/min	494 ± 2	1002 ± 52	2	0.010 (*)
	eRF1+eRF3	K_M_, nM	40 ± 7	1154 ± 318	29	0.177 (n.s.)
		v_max_, RLU/min	560 ± 20	1258 ± 257	2.2	0.223 (n.s.)

^1^ K_M_, apparent Michaelis constant; v_max_, theoretical maximal velocity. K_M_ and v_max_ were calculated from the approximation curve (Figure 5B). Values are presented as the mean ± standard error. The *p* value is shown for comparison of eRF1iso1 and corresponding eRF1iso2. n.s., non-significant difference (*p* ≥ 0.05). Asterisks indicate statistically significant differences (*, *p* < 0.05; ***, *p* < 0.001; ****, *p* < 0.0001).

## Data Availability

The data that support the findings of this study are contained within the article and the Supporting Information. All source data generated for this study are available from the corresponding author (Elena Alkalaeva; alkalaeva@eimb.ru) upon reasonable request.

## Supplementary Materials

The following supporting information can be downloaded at: https://www.mdpi.com/article/10.3390/ijms25147997/s1.
