# Supplementary material for: Functional Activity of Isoform 2 of Human eRF1
Source: Int J Mol Sci. 2024 Jul 22;25(14):7997. doi: 10.3390/ijms25147997 (PMC11277123; doi:10.3390/ijms25147997)
Supplement: Supplementary file 1 [file ijms-25-07997-s001.zip › ijms-3052234-supplementary.pdf]

eRF1iso3 – UniprotID B7Z7P8

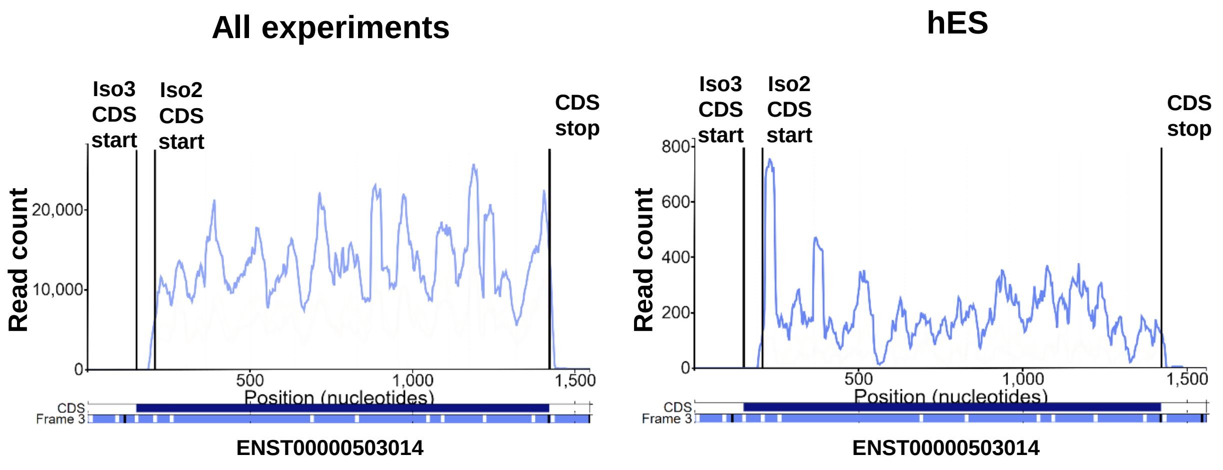

FIGURE S1. The human eRF1 isoform 3 is not translated. Visualization of ribosome profiling in the Trips-viz browser of the corresponding transcript (ENST00000503014 – iso3) among all available experiments on ribosomal profiling of human cells shows an absence of the ribosomes at the first codons of eRF1iso3. Ribosomes are present only on the part of the transcript common to all isoforms.

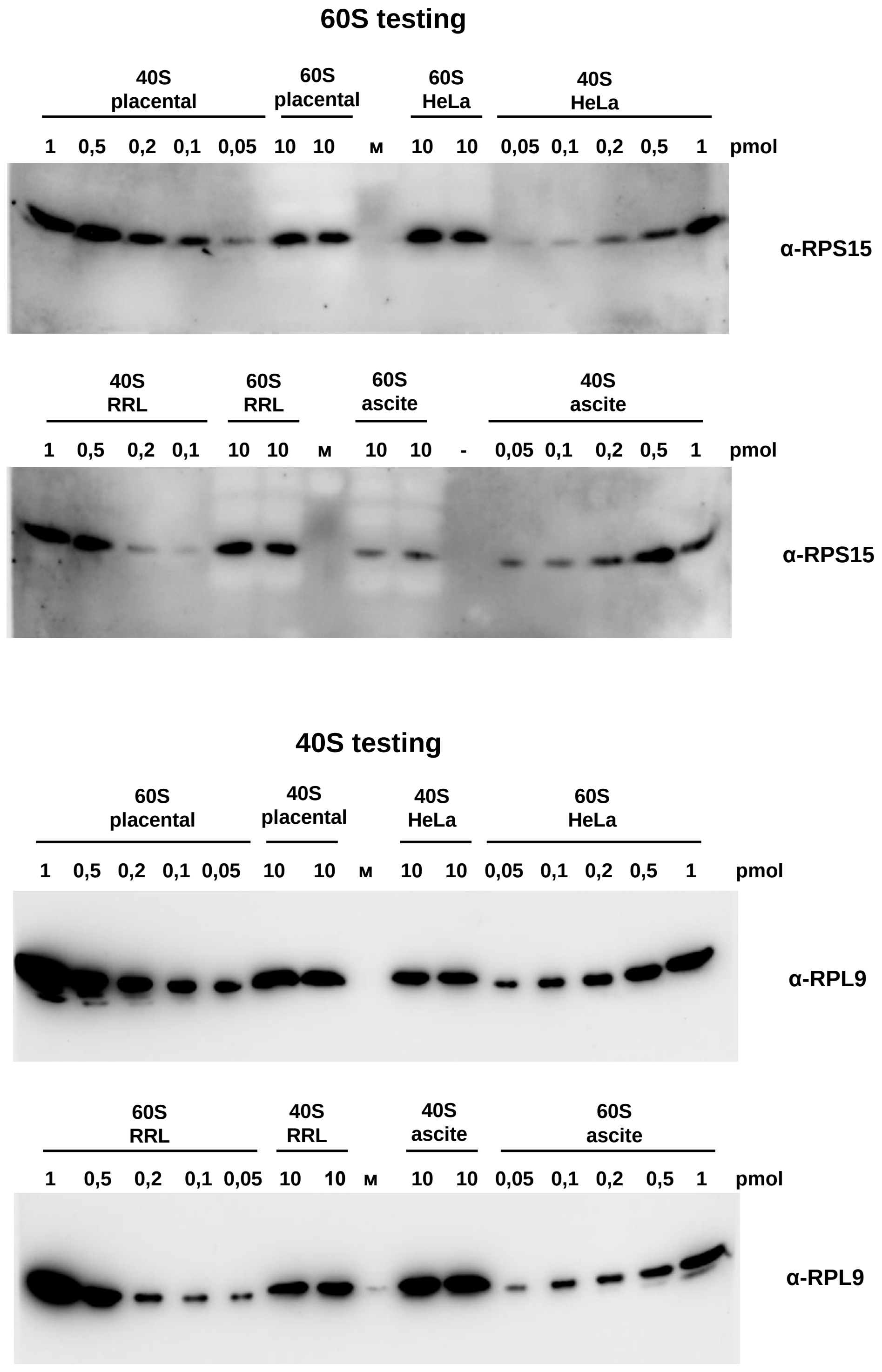

FIGURE S2. Test for cross-contamination of ribosomal subunits isolated from placenta, HeLa, RRL and Krebs-2 lysate. Antibodies raised against rpS15 and rpL9, were used for detection. An approximate estimate shows the following impurity values: 60S placental have ~2% 40S; 60S HeLa have ~10% 40S; 60S RRL have ~5% 40S; 60S ascite have ~1% 40S; 40S placental have ~2-5% 60S; 40S HeLa have ~2-5% 60S; 40S RRL have ~2-5% 60S; 40S ascite have ~10% 60S

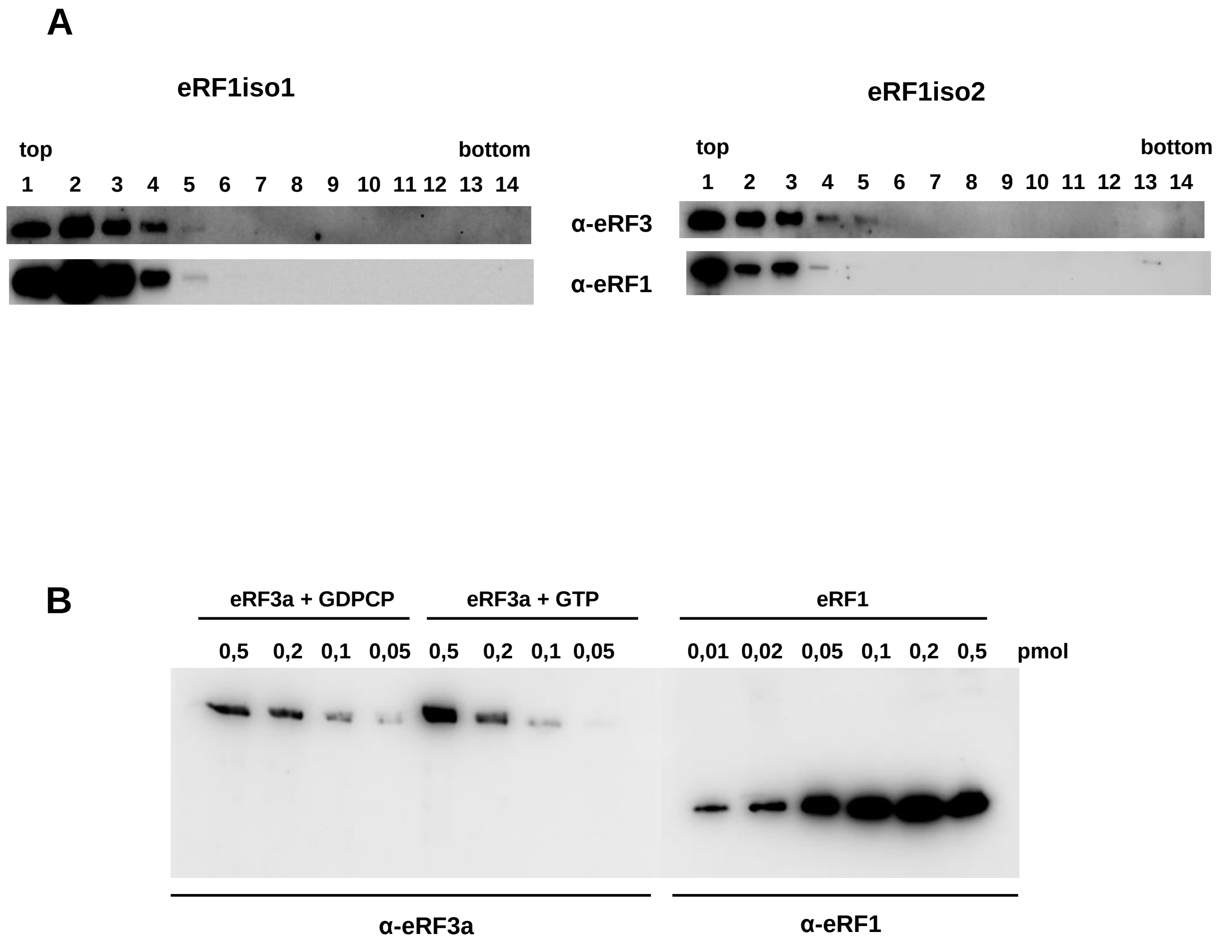

FIGURE S3. *(A)* Distribution of eRF1iso2 in the SDG. Western blot analysis of SDG of eRF1iso1, eRF1iso2 and eRF3a. The fractions of the SDG are indicated above the Western blots; fraction 1 corresponds to the top of the gradient, 14 to the bottom. *(B)* Comparison of the effectiveness of antibodies against eRF1 and eRF3a. Antibodies raised against eRF1 (sc-365686), eRF3a, were used for detection.

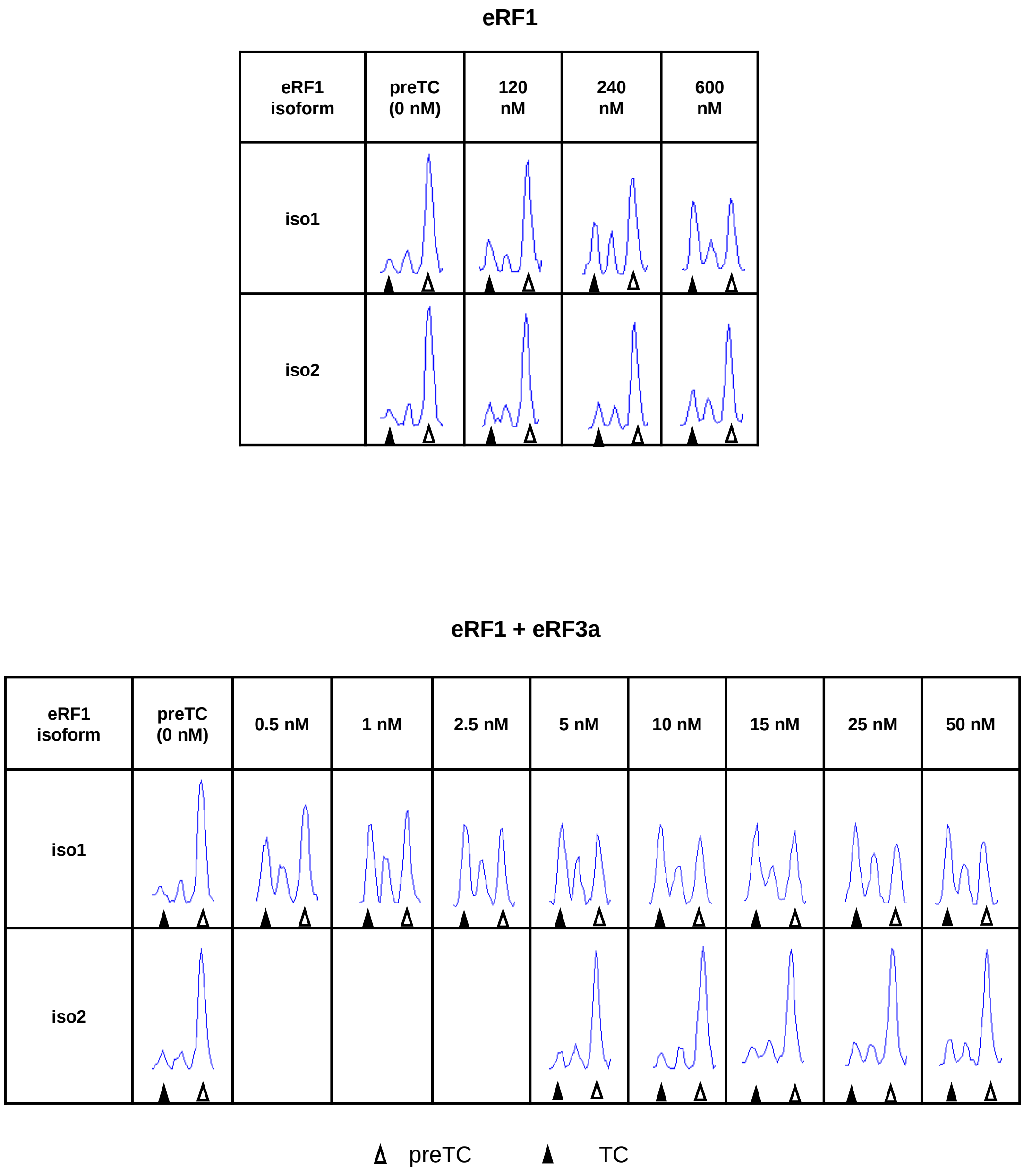

FIGURE S4. Raw toe-printing data for Figure 5A.

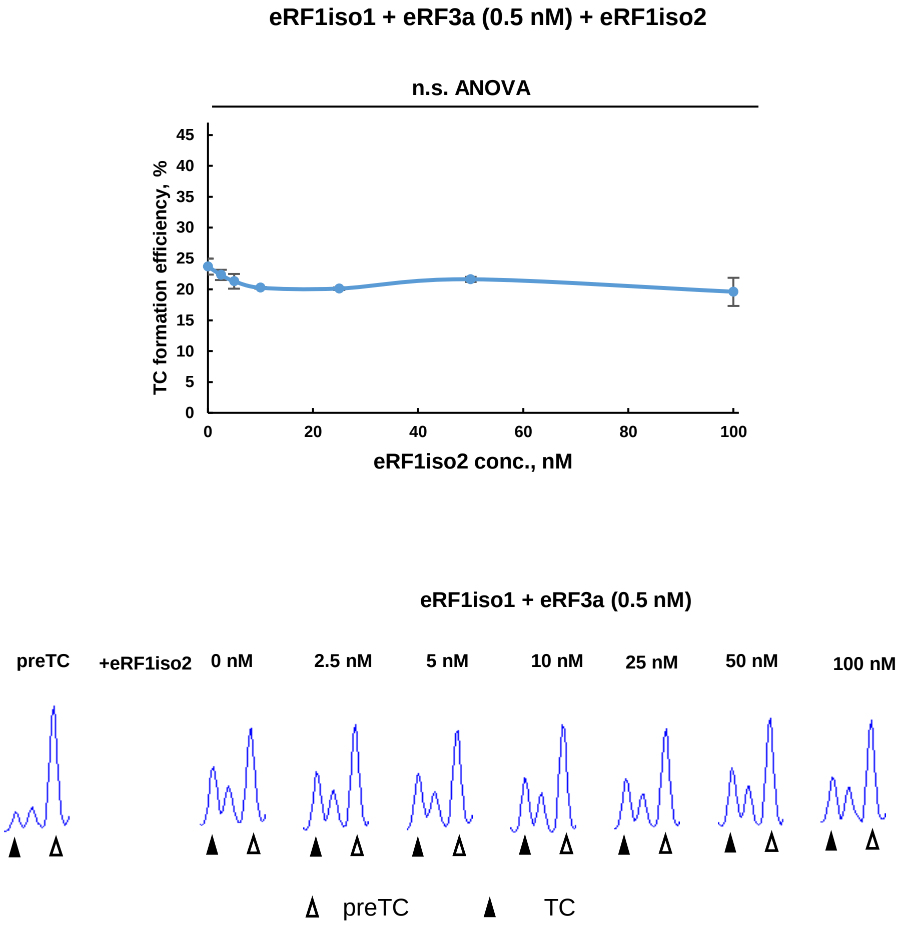

FIGURE S5. Competition of eRF1 isoforms for binding to the A site of the ribosome. Excess eRF1iso2 does not affect the efficiency of TC formation by the eRF1iso1-eRF3a complex. Toe-print analysis of TC formed at UAA stop codon in the various concentrations of eRF1 isoforms. (n=3)

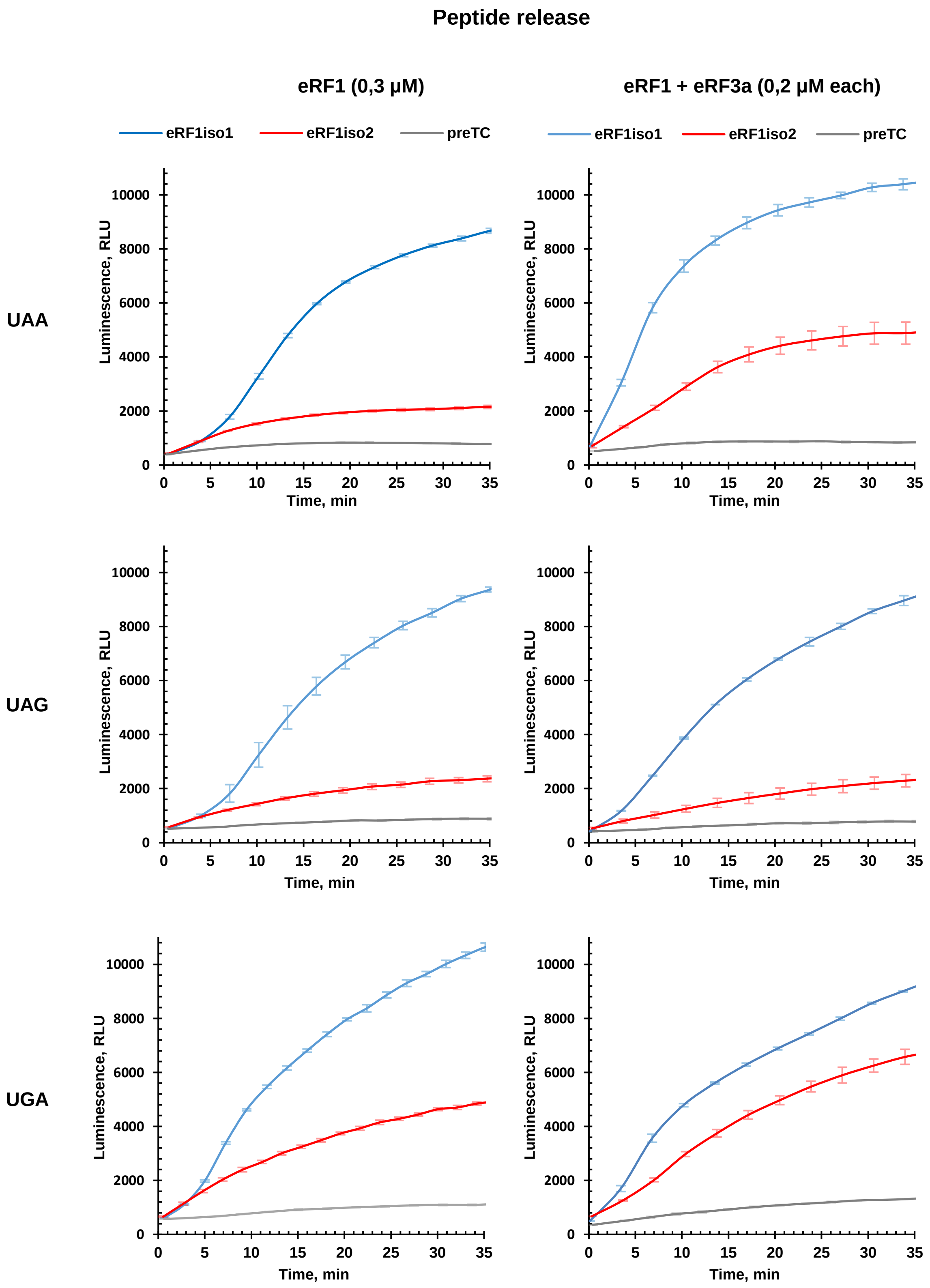

FIGURE S6. The example of the luminescence graphs showing the peptide release of Nluc, induced by eRF1 isoforms with or without eRF3a (for Figure 5B).

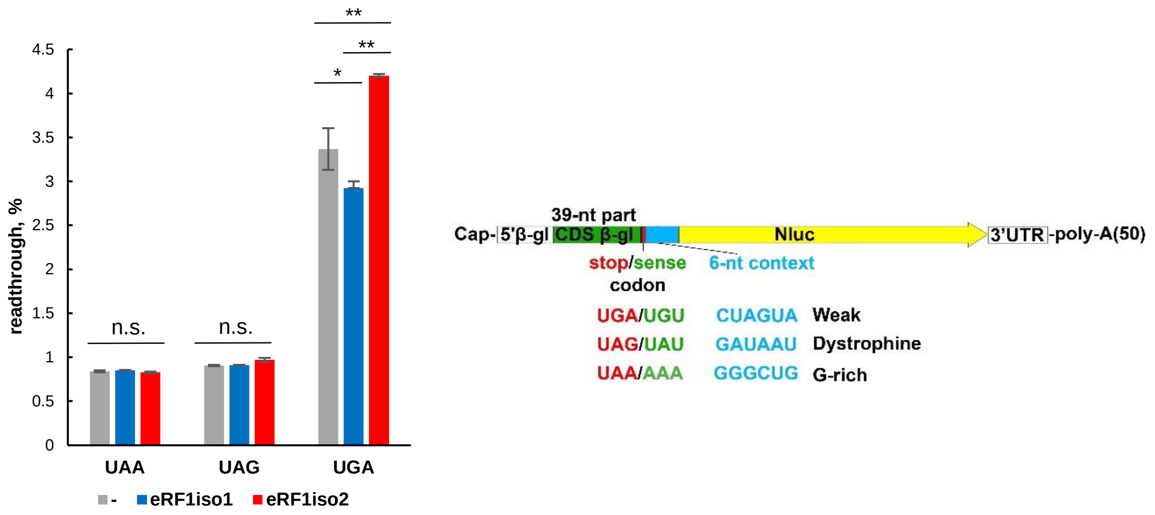

FIGURE S7. Effect of excess of eRF1 isoforms on the stop codon readthrough in HeLa lysate on Nluc reporter mRNAs with different PTCs. All experiments were carried out in three replicates. Histogram data are presented as mean relative intensity ± standard error of the mean. The difference was considered significant when P value (two-tailed t-test) was less than 0.05 (*). n.s., non-significant difference ($p \geq 0.05$). Asterisks indicate statistically significant differences (*, $p < 0.05$; **; $p < 0.01$).

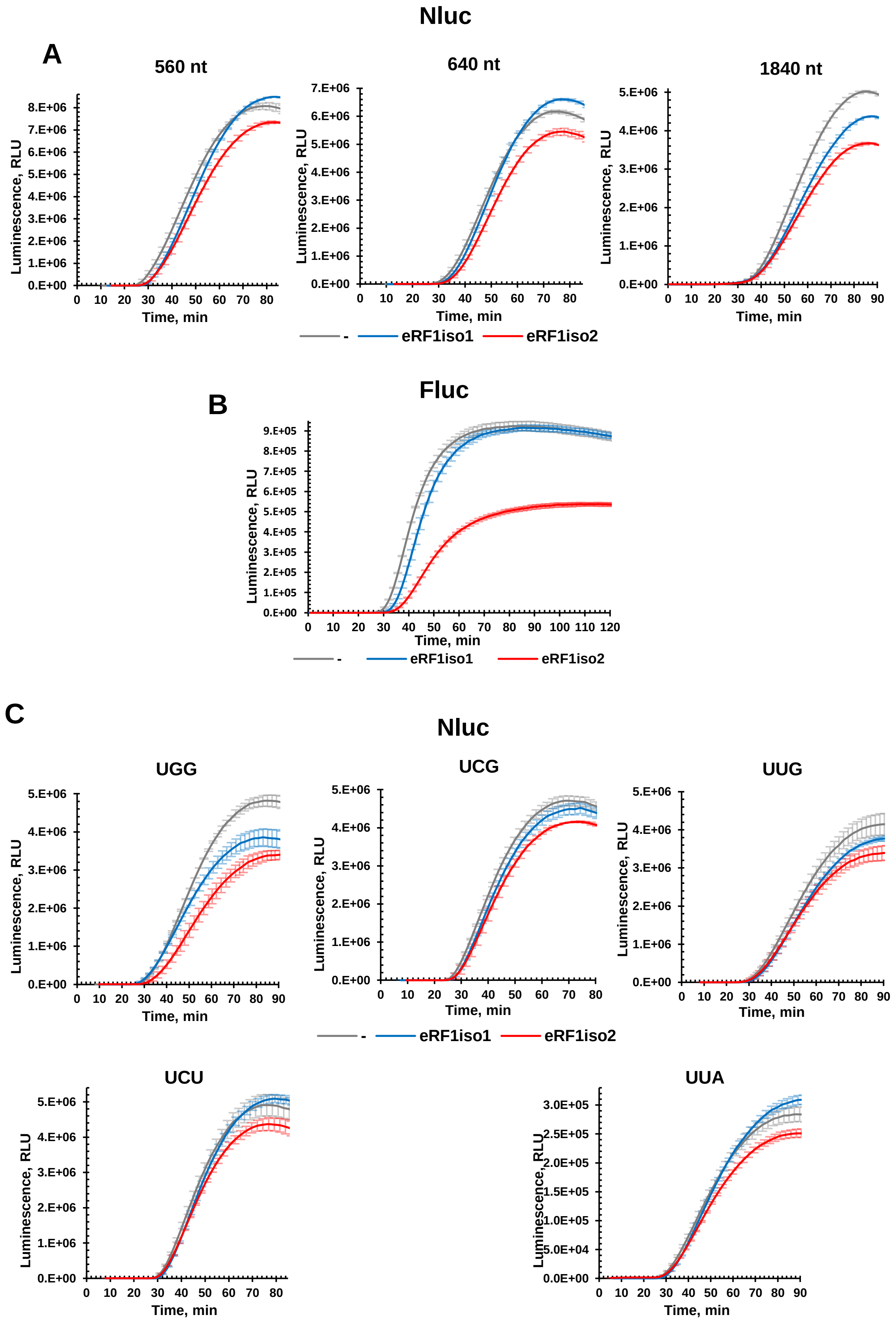

FIGURE S8. Raw data of luciferases counts for Figure 8.

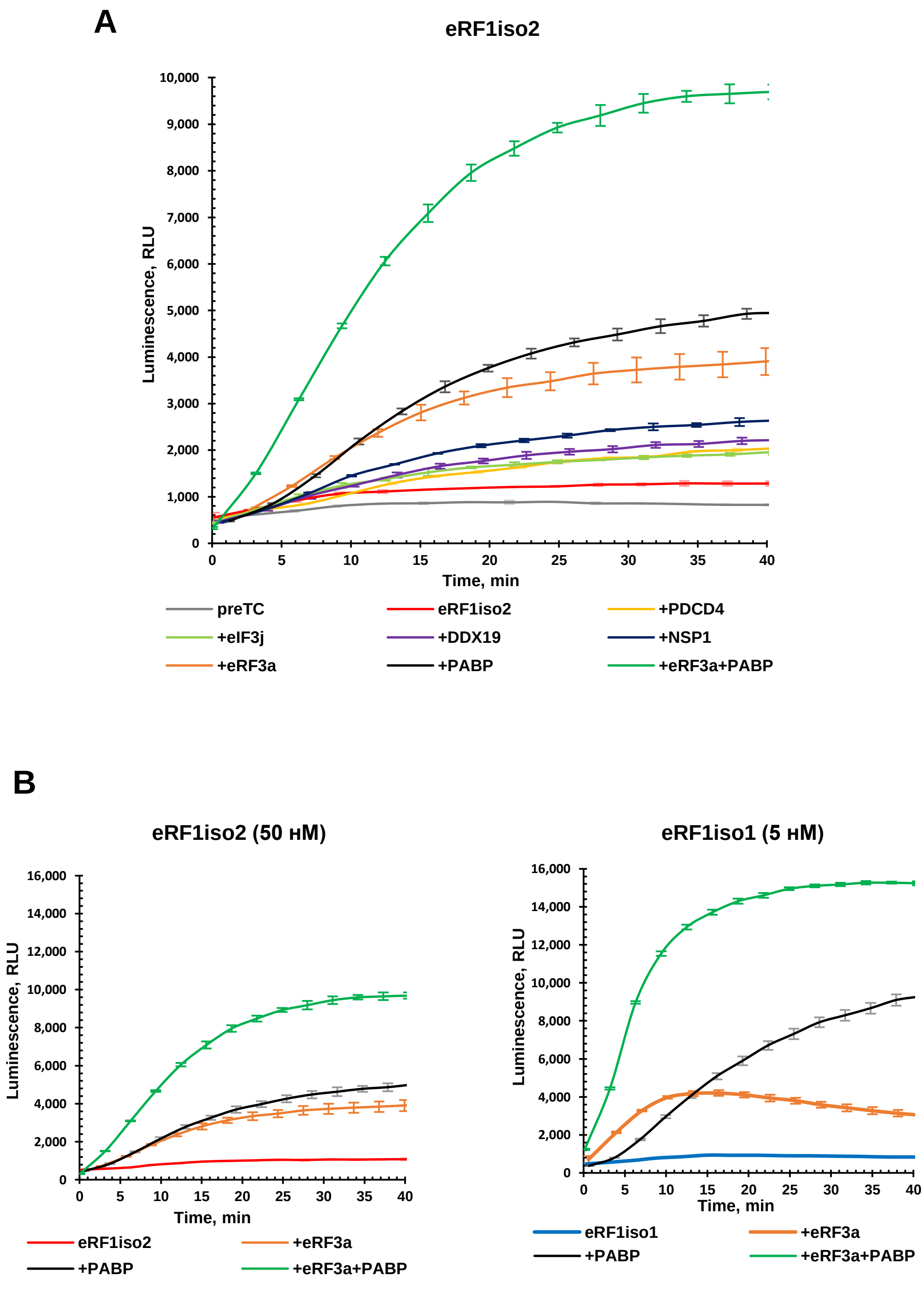

FIGURE S9. Raw luciferases counts for Figure 9

SUPPLEMENTARY METHODS

Antibodies used for Western blotting (WB), Immunocytochemistry and immunofluorescence (ICC/IF)

anti-eRF1 (Santa Cruz Biotechnology, sc-365686); anti-eRF3a (Cell Signaling, 14980S); anti-RPS15 (abcam, ab157193); anti-RPL9 (abcam, ab182556); anti-Rabbit-HRP (Jackson ImmunoResearch, 111-035-003); anti-Mouse-HRP (Jackson ImmunoResearch, 115-035-003

Plasmids

pET23_eRF1-6xHis (Table S1), encoding eRF1, was previously described (Frolova et al., 2002). PCR product 1 was obtained from the plasmid pET-6xHis-SUMO with the primers pETSUMO_F/R (Table S2). PCR products 2 and 3 were obtained from plasmid pET23_eRF1-6xHis (Table S1) using primer pairs pETSUMO_eRF1_F/R (Table S2) and pETSUMO_eRF1i2_F/pETSUMO_eRF1_R (Table S2), respectively. The plasmids pET-6xHis-SUMO-eRF1 and pET-6xHis-SUMO-eRF1iso2 were created (Table S1) from PCR products 1 and 2, 1 and 3, respectively, using the Gibson system Assembly (NEB, E2611L). The PCR product obtained from the pFastBac-Htb-GSPT1 vector (https://doi.org/10.1093/nar/gkw635) with primers pETSUMO_eRF3a_F/R (Table S2) and the PCR product 1 were used in the Gibson Assembly system (NEB, E2611L) yielded the plasmid pET-6xHis-SUMO-eRF3a (Table S1).

Plasmid pNL-gl (pl 38, table S1) was previously described in Shuvalov et al., 2021 [N]. pNL-gl was treated with the restriction enzyme NcoI and inserted into a partial CDS sequence of human β-globin with a UGA codon and Weak context (Biziaev et al. 2022) using ligation with T4 DNA ligase with primers NL-UGA_F/R (Table S2). The resulting

second start codon, ATG, was replaced by ATA using the QuikChange Site-Directed Mutagenesis Kit (Agilent) in order to obtain the pNL-UGA-Weak plasmid (Table S1). Plasmids pNL-UGU-Weak, pNL-UAA-G-rich, pNL-AAA-G-rich, pNL-UAG-Dyst, pNL-UAU-Dyst were obtained from plasmid pNL-UGA-Weak using the QuikChange Site-Directed Mutagenesis Kit (Agilent) (Table S1). Plasmids pNL-gl-UAG and pNL-gl-UGA were obtained from plasmid pNL-gl using the QuikChange Site-Directed Mutagenesis Kit (Agilent) (Table S1).

The original plasmid pGEM-Fluc (Promega, Cat.No. E1541), encoding Firefly luciferase (Fluc), contained a short 3'UTR, so we inserted a part of the 3'UTR of the GAPDH gene (which was used in (Lai et al., 2018)) 52 nt downstream from the stop codon. For this, the original vector was amplified by PCR with Q5 polymerase (NEB) using FlucGib_F/R primers (Table S2) in order to obtain PCR product 4. An insert was prepared and integrated into the linear amplified vector using a Gibson Assembly® Master Mix (NEB, E2611L) with primers FlucGN_F/R (Table S2). The resulting plasmid was named pGEM-FlucGN (Table S1). To obtain the Fluc coding sequence with premature termination codon (PTC) (Table S1), the 447th TAT codon encoding tyrosine was replaced with one of the stop codons (TAA, TAG or TGA) using the QuikChange Site-Directed Mutagenesis Kit (Agilent).

PCR product 5 was obtained from plasmid pNL-gl with the primers pNL_F/R (Table S2). PCR product 6 was obtained with the primers 5'eRF1_F/R (Table S2). PCR products 7 and 8 were obtained using the primer pairs uORF_F/R and uORF_F/uORF-ser_R (Table S2), respectively. Using the Gibson Assembly system (NEB, E2611L), plasmid pNL-full5'eRF1-Nluc (Table S1) was obtained from PCR products 5, 6 and 7, and plasmid pNL-full5'eRF1_Ser-Nluc (Table S1) was obtained from PCR products 5, 6 and 8, in which the TAG uORF stop codon was replaced by TCG encoding serine. PCR product 9 was obtained from plasmid pNL-gl with primers pNL_F and pNL-gl_R (Table S2). Using

the Gibson Assembly system (NEB, E2611L), plasmid pNL-5'β-gl-uORF-Nluc (Table S1) was obtained from PCR products 9 and 7, and plasmid pNL-5'β-gl-uORF_Ser-Nluc (Table S1) was obtained from PCR products 9 and 8, in which the TAG uORF stop codon was replaced by TCG encoding serine. PCR products 10 and 11 were obtained from the plasmid pet23_eRF1-6xHis (Table S1) with the primer pairs pNL_eRF1_F/R (Table S2), pNL_eRF1_F and pNL_eRF1-Ser_R (Table S2), respectively, using the Gibson Assembly system (NEB, E2611L). Plasmid pNL-CDSeRF1-Nluc (Table S1) was obtained from PCR products 5 and 10, and plasmid pNL-CDSeRF1-Ser-Nluc was obtained from PCR products 5 and 11 (Table S1), in which the TAG CDS stop codon of eRF1, was replaced by a TCG codon encoding serine. The constructs with the eRF1 CDS additionally contained a natural 9-nt 3′ context after the UAG stop codon.

Total RNA was isolated from HEK293 using ExtractRNA (Evrogen, BC032). Total cDNA was obtained from it using the AMV Reverse Transcription System (Promega, A3500) using Oligo(dT)15 Primer. Using primers eRF1_Bst98I (Table S2) and Oligo(dT)15, a PCR product was obtained from this cDNA, which was cloned into the TA vector pGEM®-T Easy Vector (Promega, A1360) and the resulting construct pGEM-3'eRF1 was sequenced. This product contained CDS of eRF1 and the full-length sequence of its 3′UTR, which included 861 bp after stop codon UAG and following polyA tail. It is worth to mention, that the reference mRNA sequence of eRF1 (Uniprot P62495-1, RefSeq NM_004730) includes more than 2000 bp after stop codon UAG. In order to determine whether a longer 3'UTR variant exists, we selected specific primers 3'eRF1_R1-5 (Table S2) sequentially along the entire length of 3′UTR in NM_004730. We used these primers and eRF1_Bst98I primer in PCR reaction with various conditions in order to amplify cDNA products from total RNA's of HEK293 isolated in several biological repeats. In all cases, the corresponding 3′UTR of eRF1 was synthesized only with 3'eRF1_R4-5 primers annealing the 3′UTR of eRF1 cloned sequence, including 861nt. It was not possible to

obtain products with the primers located closer to the 3' end of NM_004730 (3'eRF1_R1-3). Thus, we have obtained the sequence of 3’UTR eRF1 from HEK293.

PCR product 12 was obtained from plasmid pGEM-3'eRF1 using primers pNL_3'eRF1_F/R (Table S2). The plasmid pGEM-Fluc-3'eRF1 (Table S1) was obtained from PCR products 4 and 12 using the Gibson Assembly system (NEB, E2611L). Fluc-3'eRF1 (Table S1). Using primers NheI_F and Fluc_uORF_R (Table S2) and the plasmids pNL-full5'eRF1-Nluc and pNL-full5'eRF1-Ser-Nluc (Table S1) PCR products 13 and 14 were obtained. Using restriction enzymes *NheI* and *XbaI*, PCR product 13 was inserted into the plasmids pGEM-FlucGN and pGEM-Fluc-3'eRF1 to obtain the constructs pGEM-full5'eRF1-FlucGN and pGEM-full5'eRF1-Fluc-3'eRF1 (Table S1).

SUPPLEMENTARY TABLES

Table S1. Constructions used in this work.

№	Name	Function
1	pet23_eRF1-6xHis	bacterial expression
2	pET-6xHis-SUMO-eRF1	
3	pET-6xHis-SUMO-eRF1iso2	
4	pET-6xHis-SUMO-eRF3a	
5	pNL-UGA-Weak	For Nluc mRNA synthesis with/without PTC for readthrough studies
6	pNL-UGU-Weak	
7	pNL-UAA-G-rich	
8	pNL-AAA-G-rich	
9	pNL-UAG-Dyst	
10	pNL-UAU-Dyst	
11	pGEM-FlucGN	For Fluc mRNA synthesis with/without PTC for readthrough studies
12	pGEM-FlucGN-PTC-UAA	
13	pGEM-FlucGN-PTC-UAG	
14	pGEM-FlucGN-PTC-UGA	
15	pNL-full5’eRF1-Nluc	For Nluc mRNA synthesis with 5’UTR’s and CDS of eRF1
16	pNL-full5’eRF1-Ser-Nluc	
17	pNL-5’β-gl-uORF-Nluc	
18	pNL-5’β-gl-uORF-Ser-Nluc	
19	pNL-CDSeRF1-Nluc	
20	pNL-CDSeRF1-Ser-Nluc	
21	pGEM-full5’eRF1-FlucGN	For Fluc mRNA synthesis with the UTR’s of eRF1
22	pGEM-Fluc-3’eRF1	
23	pGEM-full5’eRF1-Fluc-3’eRF1	
25	pNL-gl	For Nluc mRNA synthesis used in Termi-Luc assay
26	pNL-gl-UAG	
27	pNL-gl-UGA	
28	pRL-TK (Promega)	For control Rluc mRNA synthesis

Table S2. Primers used in this work..

№	Name	Sequence (from 5' end)
1	petSUMO_F	AGCTTAGGTATTTATTCGGCGCAAAGTG
2	petSUMO_R	ACCACCAATCTGTTCTCTGTGAGC
3	petSUMO_eRF1_F	GAGAACAGATTGGTGGTATGGCGGACGACCCCAGT
4	petSUMO_eRF1_R	CCGAATAAATACCTAAGCTctaGTAGTCATCAAGGTCAAAAAATTCATCGTCTCCT CC
5	pETSUMO_eRF1i2_F	GAGAACAGATTGGTGGTATGATATCATTGATCATTCCTCCCAAAGACCAG
6	petSUMO_eRF3a_F	GAGAACAGATTGGTGGTATGGATCCGGGCAGTGGC
7	petSUMO_eRF3a_R	CCGAATAAATACCTAAGCTTTAGTCTTTCTCTGGAACCAGTTTCAGAACTTTTC
8	NL-UGA_F	CATGTTGCATCTGACTCCTGAGGAGAAGTCTGCCGTTACTTGACTAGTAAC
9	NL-UGA_R	CATGGTTACTAGTCAAGTAACGGCAGACTTCTCCTCAGGAGTCAGATGCAA
	FlucGib_F	CCTGGCGTTACCCAA
11	FlucGib_R	TCGGAGGATTACAATAGCTAA
12	FlucGN_F	GCTATTGTAATCCTCCGACACATTTACCCTACACATTACATTCACTTACACATTAC TTACATTCCCATTCCCATACACATCCTACATACACTAACACATACATCCCACTCTA AAATACATACAACCTG
13	FlucGN_R	GGGTAACGCCAGGTTGTATGTATTTTAGAGTGGGATGTATGTGTTAGTGTATGTA GGATGTGTATGGGAATGGGAATGTAAGTAATGTGTAAGTGAATGTAATGTGTAG GGTAAATGTGTCGG
14	pNL_F	ATAGTCTTCACACTCGAAGATTTCGTTG
15	pNL_R	CCCTATAGTGAGTCGTATTACGGC
16	5'eRF1_F	GTAATACGACTCACTATAGGGACTCGCACCGGCCCCCTCCCTTCCGCCTGCCG CCTTCCACTCCGCCCCTGGCGGAG
17	5'eRF1_R	CAGGCAGCTGCATGTGTTGCAATCCGCTCACATGGGGCCTGTGACATCACTTCC TCCGCCAGGGGCGG
18	uORF_F	ATGCAGCTGCCTGGAGAGAGGGAGCCGGTGTCCTACGTCAGAGCCGCCGCCG CCGCGGAGCCGCCGCCGGGGAGGAGCAG
19	uORF_R	CTTCGAGTGTGAAGACTATCTTCTCGCCTCCTCCTCCCTAAGGGCCCAGTCCTG GGCGGCAGCGGCTGCTCCTCCCCGGCG
20	uORF-ser_R	CTTCGAGTGTGAAGACTATCTTCTCGCCTCCTCCTCCCGAAGGGCCCAGTCCTG GGCGGCAGCGGCTGCTCCTCCCCGGCG
21	pNL-gl_R	GGTGTCTGTTTTGGGGGATTG
22	pNL_eRF1_F	CCCCAAAACAGACACCATGGCGGACGACCCCAGT
23	pNL_eRF1_R	CTTCGAGTGTGAAGACTATGTCGACTACCTAGTAGTCATCAAGGTCAAAAAATTC ATCGTCTCCTCCTTGG
24	pNL_eRF1-Ser_R	CTTCGAGTGTGAAGACTATGTCGACTACCGAGTAGTCATCAAGGTCAAAAAATTC ATCGTCTCCTCCTTGG
25	eRF1_Bst98I	CGTTTTGCCCGCTTAAGAATG

26	3'eRF1_R1	CTCTTCTCCATTTCTGTAC
27	3'eRF1_R2	CCGGTGAAGTCTCCC
28	3'eRF1_R3	CCCACCCGCCCC
29	3'eRF1_R4	CTCCCAATCCCAGC
30	3'eRF1_R5	TCACTGCGAAATGCAGC
31	pNL_3'eRF1_F	CGGAAAGTCCAAATTGTAGGTAGTCGACATGGGTC
32	pNL_3'eRF1_R	TGGGTAACGCCAGGCACTTAAATTGCATGTTTTATTTC
33	NheI_F	CGTGCTAGCCCCGCCGTAAT
34	Fluc_uORF_R	CATCCTCTAGAGGATAGAATGGCGCCGGGCCTTTCTTTATGTTTTTGGCGTCTTCCATCTTCTCGCCTCCTCC
35	Fluc_gl_ART1_koz_F	GCAGCTAATACGACTCACTATAGGCAACAACAACAACACTTGCTTTTGACACAACTGTGTTTACTTGCAATCCCCCAAAACAACGAGAATGGAAGACGCCAAAAACATAAAG
36	FlucA50_R	TTTTTTTTTTTTTTTTTTTTTTTTTTTTTTTTTTTTTTTTTTTTTTTTTTAGTTGGGTAACGCCAG
37	RV3L	CTAGCAAAATAGGCTGTCCCCAG
38	FLA50	TTTTTTTTTTTTTTTTTTTTTTTTTTTTTTTTTTTTTTTTTTTTTTTTTTAACTTGTTTATTGCAGCTTATAATGG
39	short5'eRF1_F	GCAGCTAATACGACTCACTATAGGATTGCAACACATGCAG
40	3'eRF1A50_R	TTTTTTTTTTTTTTTTTTTTTTTTTTTTTTTTTTTTTTTTTTTTTTTTTTCACTTAAATTGCATGTTTTATTTC
41	Rluc_F	GCAGCTAATACGACTCACTATAGGCTAG
42	5xTGG_Nluc_F	CCAAAACAGACACCATGTGGTGGTGGTGGTGGGTCTTCACACTCGAAGATTTCG
43	5xTCG_Nluc_F	CCAAAACAGACACCATGTCGTCGTCGTCGTCGGTCTTCACACTCGAAGATTTCG
44	5xTTG_Nluc_F	CCAAAACAGACACCATGTTGTTGTTGTTGTTGGTCTTCACACTCGAAGATTTCG
45	5xTCT_Nluc_F	CCAAAACAGACACCATGTCTTCTTCTTCTTCTGTCTTCACACTCGAAGATTTCG
46	TTA_Nluc_F	CCAAAACAGACACCATGTTATTATTATTATTAGTCTTCACACTCGAAGATTTCG
47	T7-5'gl_F	GCAGCTAATACGACTCACTATAGGGACACTTGCTTTTGACACAACTGTGTTTACTTGCAATCCCCCAAAACAGACACCATG

Table S3. mRNA constructions used in the work.

№	Name	Template from table 1	Primers from table 2	Working concentration in translation *in vitro*, nM	Function
1	Fluc	11	35, 36	40	PTC readthrough
2	Fluc_PTC_UAA	12	35, 36	40	
3	Fluc_PTC_UAG	13	35, 36	40	
4	Fluc_PTC_UGA	14	35, 36	40	
5	NL-UGA-Weak	5	37, 38	2	
6	NL-UGU-Weak	6	37, 38	2	
7	NL-UAA-G-rich	7	37, 38	2	
8	NL-AAA-G-rich	8	37, 38	2	
9	NL-UAG-Dyst	9	37, 38	2	
10	NL-UAU-Dyst	10	37, 38	2	
11	5'eRF1-Fluc-3'GAPDH	21	39, 48	40	Influence of eRF1 UTR's on translation
12	5'β-gl-Fluc-3'eRF1	22	35, 40	40	
13	5'eRF1-Fluc-3'eRF1	23	39, 40	40	
14	full5'eRF1-UAG-Nluc	15	37, 38	15	Stop codons UAG readthrough of eRF1 uORF and CDS
15	full5'eRF1-UCG-Nluc	16	37, 38	15	
16	5'eRF1-UAG-Nluc	15	39, 38	15	
17	5'eRF1-UCG-Nluc	16	39, 38	15	
18	5'β-gl-uORF-UAG-Nluc	17	37, 38	1.5	
19	5'β-gl-uORF-UCG-Nluc	18	37, 38	1.5	
20	CDSeRF1-Nluc-UAG	19	37, 38	10	
21	CDSeRF1-Nluc-UCG	20	37, 38	10	
22	Nluc-UAA	25	37, 38	-	For Termi-Luc assay
23	Nluc-UAG	26	37, 38	-	
24	Nluc-UGA	27	37, 38	-	
25	Rluc	28	41, 38	-	The control for mRNA transfection efficiency
27	5xUGG_Nluc_F	25	47, 42, 38	2	Translation of Nluc mRNAs with additional sense coodns in the presence of eRF1 isoforms in HEK293 lysate.
28	5xUCG_Nluc_F	25	47, 43, 38	2	
29	5xUUG_Nluc_F	25	47, 44, 38	2	
30	5xUCU_Nluc_F	25	47, 45, 38	2	
31	5xUUA_Nluc_F	25	47, 46, 38	2	

3′UTR sequence of eRF1 (Bold) cloned from Hek293 after the stop codon *TAG* of eRF1 CDS (underlined)

CGTTTTGCCCGCTTAAGAATGGAAAAGCGACATAACTATGTTCGGAAAGTAGCAGAGACTG
CTGTGCAGCTGTTTATTTCTGGGGACAAAGTGAATGTGGCTGGTCTAGTTTTAGCTGGATC
CGCTGACTTTAAAACTGAACTAAGTCAATCTGATATGTTTGATCAGAGGTTACAATCAAAAG
TTTTAAAATTAGTTGATATATCCTATGGTGGTGAAAATGGATTCAACCAAGCTATTGAGTTAT
CTACTGAAGTCCTCTCCAACGTGAAATTCATTCAAGAGAAGAAATTAATAGGACGATACTTT
GATGAAATCAGCCAGGACACGGGCAAGTACTGTTTTGGCGTTGAAGATACACTAAAGGCT
TTGGAAATGGGAGCTGTAGAAATTCTAATAGTCTATGAAAATCTGGATATAATGAGATATGT
TCTTCATTGCCAAGGCACAGAAGAGGAGAAAATTCTCTATCTAACTCCAGAGCAAGAAAAG
GATAAATCTCATTTCACAGACAAAGAGACCGGACAGGAACATGAGCTTATCGAGAGCATGC
CCCTGTTGGAATGGTTTGCTAACAACTATAAAAAATTTGGAGCTACGTTGGAAATTGTCACA
GATAAATCACAAGAAGGGTCTCAGTTTGTGAAAGGATTTGGTGGAATTGGAGGTATCTTGC
GGTACCGAGTAGATTTCCAGGGAATGGAATACCAAGGAGGAGACGATGAATTTTTTGACCT
TGATGACTAC*TAG***GTAGTCGACATGGGTCCGGCAAAACGTGCCTCACCCTCCAGCATCC
AACCCAAGGAGCATACCCATGGTGGAATCCAAACAGATCCCTGCCTTACAATTGGAACA
TTTCCAGAACTTAATCCATGAGCATTGGATATTGAAAAGAAAACCGAAACAAAACCAGA
CCCAGCCCTACACTTTGGTTTGTCATGGTGTCAGCGCAGCAGCCTACAACTAAGTTCCT
AAACGCCACTTTGGACTAATTTAAAAAAGAATCCCAGTTTTTACTTTTACTGGATGGTGA
AATTGGTTGCTCTTGTATTTTATGAAAAAAAATGATTTTTTTAACCTTCATACATAGAAGC
AAAAATACTTTAACTGCTGTAAACCTTCAAAAGTTAATAGAAGTGAGATCATACTGGTTT
GTTTCTTATTTTGATTGGAGAAAAATTAAATTGCTGCATTTCGCAGTGACCCATTTACATG
GCATTCTCAGCTTAGACTGCGTAAGAAGAAATATATGTGGTGAAATGTTGGAACCATTTC
TCTCTTGGTCTCTGTTTAATGTTGAAAGGGTGAGCTAATAGGAGGCACTTTCAACTTCAC
TCCCTCACGCTACCCCGTCCCCCTCCAGACTGGCAGTTTCAAGGATGCAAATTGCATTG
CAAAATCAAACTGACTCATGAAGCATTTGGGCCAGTGCACTGTTTACTTCCATCTGTTTG
CAGACACATTTGTGCCCGGCGTTTGGGAGCCCTTTGTATCAATGTTCTGACAAGGGTCC
CTATAACCTTAACCTACTCGAAACCGGTTTGGGATGGATATGATGGGGCTTCTGTGCTAT
TGCTGGGATTGGGAGAAATAAAACATGCAATTTAAGTGCAAAAAAAAAAAAAAAAAAAA
AAAAAAAAA**
